# Inhibition of PRKAA/AMPK (Ser485/491) phosphorylation by crizotinib induces cardiotoxicity via perturbing autophagosome-lysosome fusion

**DOI:** 10.1080/15548627.2023.2259216

**Published:** 2023-09-21

**Authors:** Zhifei Xu, Zezheng Pan, Ying Jin, Zizheng Gao, Feng Jiang, Huangxi Fu, Xueqin Chen, Xiaochen Zhang, Hao Yan, Xiaochun Yang, Bo Yang, Qiaojun He, Peihua Luo

**Affiliations:** a Center for Drug Safety Evaluation and Research of Zhejiang University, College of Pharmaceutical Sciences, Zhejiang University, Hangzhou, Zhejiang, P.R.China; b Department of Oncology, Affiliated Hangzhou Cancer Hospital, Zhejiang University School of Medicine, Hangzhou, Zhejiang, P.R.China; c Cancer Center, Zhejiang University, Hangzhou, Zhejiang, P.R.China; d Department of Medical Oncology, The First Affiliated Hospital, Zhejiang University School of Medicine, Hangzhou, Zhejiang, P.R.China; e Institute of Pharmacology & Toxicology, College of Pharmaceutical Sciences, Zhejiang University, Hangzhou, Zhejiang, P.R.China; f Department of Cardiology, Second Affiliated Hospital, School of Medicine, Zhejiang University, Hangzhou, Zhejiang, P.R.China; g Deparment of Pharmaceutical and Translational Toxicology, Innovation Institute for Artificial Intelligence in Medicine of Zhejiang University, Hangzhou, Zhejiang, P.R.China; h Department of Pharmacology and Toxicology, Hangzhou Institute of Innovative Medicine, College of Pharmaceutical Sciences, Zhejiang University, Hangzhou, Zhejiang, P.R.China

**Keywords:** Autophagosome-lysosome fusion, autophagy, cardiotoxicity, crizotinib, metformin, PRKAA/AMPK

## Abstract

Crizotinib, a small-molecule tyrosine kinase inhibitor targeting ALK, MET and ROS1, is the first-line drug for ALK-positive metastatic non-small cell lung cancer and is associated with severe, sometimes fatal, cases of cardiac failure, which increases the risk of mortality. However, the underlying mechanism remains unclear, which causes the lack of therapeutic strategy. We established in vitro and in vivo models for crizotinib-induced cardiotoxicity and found that crizotinib caused left ventricular dysfunction, myocardial injury and pathological remodeling in mice and induced cardiomyocyte apoptosis and mitochondrial injury. In addition, we found that crizotinib prevented the degradation of MET protein by interrupting autophagosome-lysosome fusion and silence of MET or re-activating macroautophagy/autophagy flux rescued the cardiomyocytes death and mitochondrial injury caused by crizotinib, suggesting that impaired autophagy activity is the key reason for crizotinib-induced cardiotoxicity. We further confirmed that recovering the phosphorylation of PRKAA/AMPK (Ser485/491) by metformin re-activated autophagy flux in cardiomyocytes and metformin rescued crizotinib-induced cardiomyocyte injury and cardiac complications. In summary, we revealed a novel mechanism for crizotinib-induced cardiotoxicity, wherein the crizotinib-impaired autophagy process causes cardiomyocyte death and cardiac injury by inhibiting the degradation of MET protein, demonstrated a new function of impeded autophagosome-lysosome fusion in drugs-induced cardiotoxicity, pointed out the essential role of the phosphorylation of PRKAA (Ser485/491) in autophagosome-lysosome fusion and confirmed metformin as a potential therapeutic strategy for crizotinib-induced cardiotoxicity.

**Abbreviations and Acronyms:** AAV: adeno-associated virus; ACAC/ACC: acetyl-Co A carboxylase; AMP: adenosine monophosphate; AMPK: AMP-activated protein kinase; ATG5: autophagy related 5; ATG7: autophagy related 7; CHX: cycloheximide; CKMB: creatine kinase myocardial band; CQ: chloroquine; c-PARP: cleaved poly (ADP-ribose) polymerase; DAPI: 4ʹ6-diamidino-2-phenylindole; EF: ejection fraction; FOXO: forkhead box O; FS: fractional shortening; GSEA: gene set enrichment analysis; H&E: hematoxylin and eosin; HF: heart failure; HW: TL: ratio of heart weight to tibia length; IR: ischemia-reperfusion; KEGG: Kyoto encyclopedia of genes and genomes; LAMP2: lysosomal-associated membrane protein 2; LDH: lactate dehydrogenase; MCMs: mouse cardiomyocytes; MMP: mitochondrial membrane potential; mtDNA: mitochondrial DNA; MYH6: myosin, heavy peptide 6, cardiac muscle, alpha; MYH7: myosin, heavy peptide 7, cardiac muscle, beta; NPPA: natriuretic peptide type A; NPPB: natriuretic peptide type B; PI: propidium iodide; PI3K: phosphoinositide 3-kinase; PRKAA/AMPKα: protein kinase AMP-activated catalytic subunit alpha; qPCR: quantitative real-time PCR; SD: standard deviation; SRB: sulforhodamine B; TKI: tyrosine kinase inhibitor; WGA: wheat germ agglutinin

## Introduction

Crizotinib, a tyrosine kinase inhibitor (TKI) targeting ALK, MET and ROS1, was approved as the first-line drug for ALK-rearranged metastatic non-small cell lung cancer [1], as ALK rearrangement is known as “diamond mutation” and lead to high sensitivity to ALK TKIs [2]. The remarkable therapeutic efficacy of crizotinib makes ALK-rearranged non-small cell lung cancer a manageable chronic disease and extended lifespan of these patients. However, severe cardiotoxicity, including cardiac dysfunction, has been reported in patients treated with crizotinib, which leads to poor prognosis, even 25% fatality in patients after cardiac adverse effects [3] and has been paid substantial attention by physicians, researchers and official drug-administration agencies. In the past decade, crizotinib-induced cardiac dysfunction has been proved to be associated with cardiomyocyte death [4]. However, the underlying molecular mechanisms for crizotinib-induced cardiomyocyte death and cardiac dysfunction are still poorly understood, as well as the effective management or intervention strategies to improve its safety and clinical application.

Macroautophagy/autophagy is an evolutionarily conserved process that is closely related to cardiomyocyte death and survival. Impaired autophagy activity was observed during cardiac diseases, including ischemia-reperfusion (IR) injury, myocardial infarction, cardiac hypertrophy, and heart failure (HF) [5–7]. Indeed, it has been reported that knockdown of *Atg5* (autophagy related 5), *Atg7* (autophagy related 7), and *Becn1* (beclin 1), genes essential for autophagy, lead to the defects in cardiac morphogenesis and structure, and reduced survival in zebrafish [8]. In addition, cardiomyocyte-specific deletion of *Atg5* caused cardiac hypertrophy and dysfunction [9], and cardiomyocyte-specific deletion of *Atg7* caused cardiac systolic dysfunction [10] and reduced the survival of mice [11]. Typically, the inhibition of autophagy process is the key reason for death of cardiomyocytes in cardiac dysfunction and remodeling [9,12]. Crizotinib has been found to be related to blockage of autophagy flux and subsequent death of pulmonary artery smooth muscle cells [13] and our preliminary data showed that crizotinib could inhibit autophagosome-lysosome fusion in cardiomyocytes. Thus, the study on the role of impaired autophagosome-lysosome fusion in crizotinib-induced cardiomyocyte death and its regulatory mechanism would benefit the identification of the key mechanism and therapeutic targets for cardiac dysfunction caused by crizotinib.

AMP-activated protein kinase (AMPK), one of the most important master regulator of energy metabolic homeostasis, could maintain cardiac function [14,15], prevent cardiac hypertrophy and HF [16–18] and orchestrates autophagy processes, including autophagosome formation [19,20] and autophagosome-lysosome fusion [21,22]. Thus, AMPK inhibition might cause reduced autophagy activity and cardiac injury or diseases. In particular, the phosphorylation of PRKAA/AMPKα (protein kinase AMP-activated catalytic subunit alpha) at Thr172 has been proved to be required for autophagosome formation. However, the essential residue required for autophagosome-lysosome fusion is much less known. Cadmium, which could inhibit the phosphorylation of serine 485 residue [23], caused defective autophagosome-lysosome fusion [24], promoting the hypothesis that serine 485 might be the key residue within PRKAA that is required for autophagosome-lysosome fusion.

In this study, we revealed a novel mechanism for crizotinib-induced cardiotoxicity, wherein the crizotinib-impaired autophagy process causes cardiomyocyte death and cardiac injury by inhibiting the degradation of MET protein, pointing out the new function of impeded autophagosome-lysosome fusion in drugs-induced cardiotoxicity. In addition, we confirmed that the phosphorylation of PRKAA (Ser485/491) is required for autophagosome-lysosome fusion and cardiac function and recovering PRKAA (Ser485/491) phosphorylation by metformin is a potential therapeutic strategy for crizotinib-induced cardiotoxicity.

## Results

### Crizotinib causes cardiac complications in mice and cardiomyocyte apoptosis

**Figure 1.**
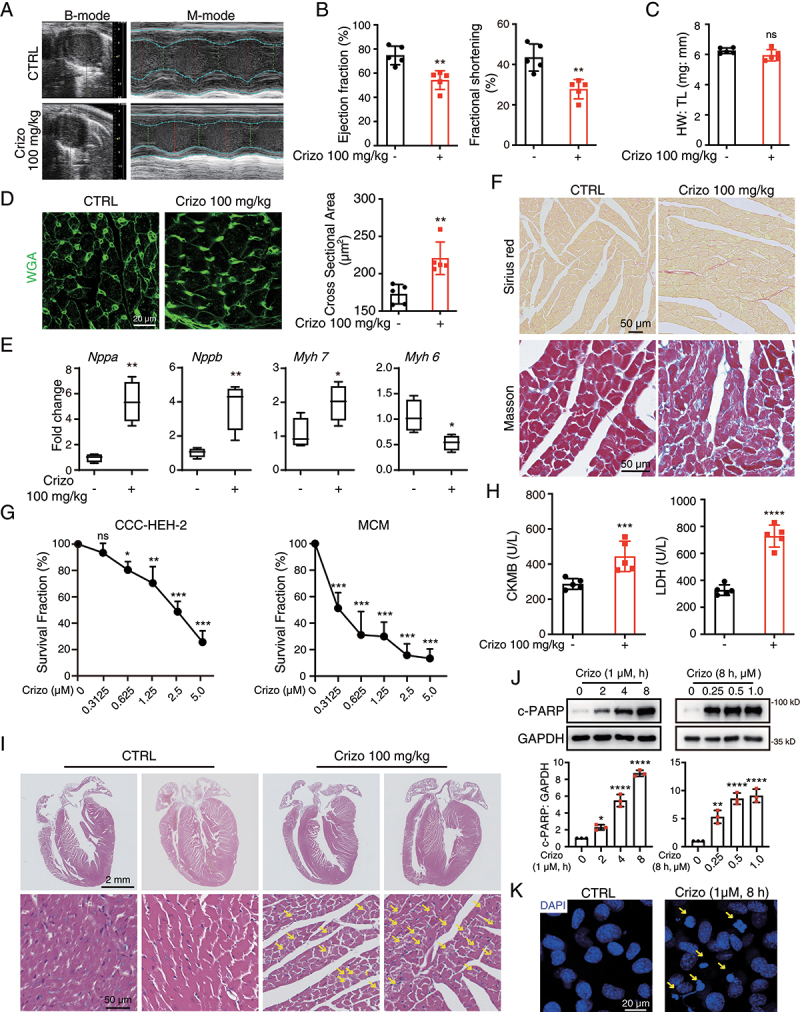
Crizotinib causes cardiac dysfunction, myocardial injury and remodeling in mice. C57BL/6J mice (*n* = 5) were treated with vehicle or 100 mg/kg crizotinib for 6 weeks. (A, B) the cardiac function of C57BL/6J mice. (C) heart weight to tibia length ratio (HW: TL). (D) Representative images of cardiac sections stained by WGA. Scale bar: 20 μm. Right: quantification of cardiomyocyte cross-sectional area based on WGA staining. (E) cardiac remodeling gene expression relative to *Actb*. *n* = 4. (F) Representative images of cardiac sections stained by Sirius red or Masson. Scale bar: 50 μm. (G) MCMs (left) or CCC-HEH-2 cells (right) were treated with crizotinib for 72 h, respectively, and the survival fraction was detected via SRB assay. *n* = 3. (H) serum from the mice was analyzed for CKMB (left) and LDH (right) levels. *n* = 5. (I) H&E staining. Scale bar: 2 μm. The yellow arrow indicated typical pathological changes. Scale bar: 50 μm. (J) MCMs were treated with crizotinib. GAPDH was used as the loading control. *n* = 3. (K) MCMs were stained with DAPI after crizotinib (1 μM) treatment for 8 h. Yellow arrows indicate significant changes of the nucleus. Scale bar: 25 μm. Data were presented as mean ± SD. The *P* value was calculated by unpaired Student’s t test (B to E and H) and one-way ANOVA with Dunnett’s multiple comparisons tests (G and J). ****, *P* < 0.0001; ***, *P* < 0.001; **, *P* < 0.01; *, *P* < 0.05; ns, no significance. CTRL: Control; Crizo: crizotinib.

We first established a mouse model for cardiac complications caused by crizotinib. 6-week treatment of crizotinib significantly reduced the cardiac ejection fraction (EF) and fractional shortening (FS) of mice (Figure 1A, B), suggesting that crizotinib caused cardiac dysfunction in mice, which was consistent with the clinical observations of crizotinib-induced cardiac complications. We found that crizotinib showed little effect on the ratio of heart weight to tibia length (HW: TL) in mice (Figure 1C). Wheat germ agglutinin (WGA) staining showed increased cardiomyocyte size in crizotinib-treated hearts (Figure 1D), suggesting that crizotinib caused cardiac hypertrophy. Meanwhile, quantitative real-time PCR (qPCR) analysis on the expression of markers for pathological cardiac remodeling [25,26] showed that crizotinib significantly increased mRNA expression of *Nppa*, *Nppb* (natriuretic peptide type A, B), and *Myh*7 (myosin, heavy peptide 7, cardiac muscle, beta), while significantly decreasing mRNA expression of *Myh6* (myosin, heavy peptide 6, cardiac muscle, alpha) in hearts (Figure 1E), suggesting that crizotinib caused pathological remodeling in hearts. Notably, crizotinib caused cardiac fibrosis as Sirius red and Masson trichromatic staining revealed collagen deposition in crizotinib-treated hearts (Figure 1F). These results demonstrated that crizotinib induced cardiac dysfunction and pathological remodeling in mice, which phenocopies the clinical observations of crizotinib-induced cardiac complications.

It has been reported that cardiac pathological remodeling is attributable to cardiomyocyte death due to its limited regeneration capacity [27,28] and a study revealed that crizotinib might be associated with cardiomyocyte death [4]. Therefore, we then investigated the effect of crizotinib on cardiomyocyte survival. Sulforhodamine B (SRB) assay showed that the survival fraction of CCC-HEH-2, a human embryonic cardiac tissue-derived cell line, and primary adult mouse cardiomyocytes (MCMs) significantly reduced upon crizotinib treatment (Figure 1G). Moreover, elevated serum creatine kinase myocardial band (CKMB) and LDH (lactate dehydrogenase) levels were detected in blood from crizotinib-treated mice (Figure 1H), indicating the myocardial damage. Histological analysis by hematoxylin and eosin (H&E) staining revealed diffuse and vacuolated cytoplasmic lesions and decreased myocardial cell density in crizotinib-treated mice hearts (Figure 1I), further supporting the existence of cardiomyocyte death and myocardial injury. Western blot results showed increased expression of c-PARP (cleaved poly (ADP-ribose) polymerase), a marker for apoptosis, in crizotinib-treated MCMs (Figure 1J) and CCC-HEH-2 cells (Figure S1A and S1B). Flow cytometry with propidium iodide (PI) and ANXA5/Annexin V co-staining further confirmed that crizotinib increased the apoptosis rate of cardiomyocytes (Figure S1C). Apoptotic cells were detected in heart sections from crizotinib-treated mice by TUNEL staining (Figure S1D). 4ʹ6-diamidino-2-phenylindole (DAPI) staining of the nuclei showed pyknosis in crizotinib-treated MCMs (Figure 1K), suggesting that DNA damage might exist. Indeed, γ-H2AX, a marker for DNA damage, increased dramatically in CCC-HEH-2 cells (Figure S1E) and MCMs (Figure S1F) after crizotinib treatment, which was confirmed by immunofluorescence assay in heart sections (Figure S1G). Taken together, our results confirmed that cardiomyocyte apoptosis could be the cause of cardiac pathological remodeling and dysfunction caused by crizotinib.

### Crizotinib induces mitochondrial injury in cardiomyocytes

It has been well-defined that mitochondrial dysfunction is closely associated with cardiomyocyte death [29–31] and heart failure [32,33]. In addition, the mitochondrial pathway is one of the main signaling pathways that contribute to apoptosis [34]. We found that crizotinib apparently increased the expression of BAX (BCL2 associated X, apoptosis regulator) and CYCS (cytochrome c, somatic), two effectors of mitochondrial apoptosis (Figure S2A), suggesting the activation of mitochondrial apoptotic signaling. Therefore, to elucidate the mechanism for crizotinib-induced cardiomyocyte apoptosis, we then investigated the effect of crizotinib on mitochondria. The expression of HSPD1/HSP60 (a mitochondrial matrix protein) and TOMM20 (translocase of outer mitochondrial membrane 20; a mitochondrial membrane protein) decreased after crizotinib treatment (Figure S2B and S2C). Flow cytometry with JC-1 staining showed that crizotinib induced decrease in mitochondrial membrane potential (MMP) of cardiomyocytes (Figure S2D and S2E), indicating mitochondrial damage. We then detected the ultrastructure of cardiac ventricular tissue of mice by transmission electron microscopy (TEM) and found disorganized mitochondrial cristae in cardiac ventricular tissues from crizotinib-treated mice, which was manifested as vacuole-like swelling and reduced mitochondrial matrix density (Figure S2F and S2G). Mitochondrial DNA (mtDNA) exists independently of the nucleus and is an essential marker for mitochondrial function, which could be reflected by the relative expression level of *MT-ATP6* (mitochondrially encoded ATP synthase membrane subunit 6) and *RPL13* (ribosomal protein L13; a genomic gene) genes (*MT-ATP6*: *RPL13*). We found that the mtDNA level decreased significantly after crizotinib treatment in heart tissues and CCC-HEH-2 cells respectively (Figure S2H and S2I). These results suggested that crizotinib could induce mitochondrial damage, which leads to cardiomyocyte apoptosis.

### Accumulated MET protein contributes to crizotinib-induced cardiomyocyte death and cardiac complication

**Figure 2.**
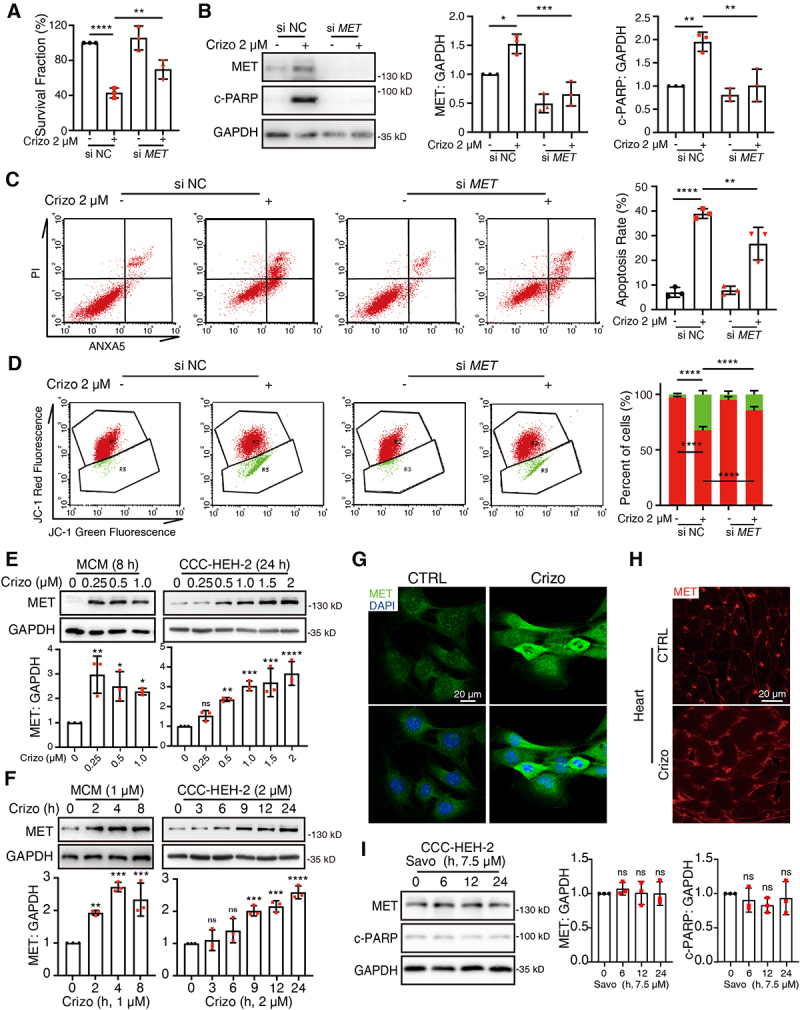
The accumulation of MET protein leads to cardiomyocyte injury. (A) CCC-HEH-2 cells were transfected with siRNA targeting MET, and then treated with or without crizotinib for 72 h. The survival fraction was detected. *n* = 3. (B) CCC-HEH-2 cells were treated with crizotinib for 24 h after transfection. Representative immunoblots (left) and relative quantification (right) of the MET and c-PARP were shown. GAPDH was used as a loading control. *n* = 3. (C) CCC-HEH-2 cells were treated with crizotinib for 36 h after transfection. Apoptosis rates were detected by PI and ANXA5 co-staining and flow cytometry. Representative images (left) and relative quantification (right) were shown. *n* = 3. (D) CCC-HEH-2 cells were treated with crizotinib for 24 h after siRNA transfection. Then, cells were stained with JC-1. Relative mean fluorescence values of red and green were detected by flow cytometry. Representative images were shown (left) and a statistical histogram was presented (right). *n* = 4. (E, F) MCMs and CCC-HEH-2 cells were treated with crizotinib as indicated. GAPDH was used as a loading control. The quantification was shown beneath the immunoblots. *n* = 3. (G) MET staining in MCM cells treated with 1 μM crizotinib for 8 h. Scale bar: 20 μm. (H) MET staining from cardiac sections of mice. Scale bar: 20 μm. (I) CCC-HEH-2 cells were treated with savolitinib. Representative immunoblot (left) and relative quantification (right) of MET and c-PARP were shown. *n* = 3. Data were presented as mean ± SD. The *P* value was calculated by one-way ANOVA with Sidak’s (A to D) test and Dunnett’s (E, F and I) multiple comparisons test. ****, *P* < 0.0001; ***, *P* < 0.001; **, *P* < 0.01; *, *P* < 0.05; ns, no significance. CTRL: control; Crizo: crizotinib; Savo: savolitinib.

As a multi-target TKI, crizotinib targets ALK, MET and ROS1. We sought to investigate whether the cardiotoxic effect of crizotinib is caused by the inhibition of these kinases. As ALK or ROS1 fusion proteins are mainly expressed in cancer cells, we mainly focused on MET kinase. We found that silence of the *MET* gene rescued crizotinib-reduced survival of cardiomyocytes (Figure 2A). Crizotinib-increased c-PARP protein level and apoptosis rate in cardiomyocytes were reversed by *MET* silence (Figure 2B, C). In addition, the silence of the *MET* gene also rescued crizotinib-decreased MMP (Figure 2D). Interestingly, we found that crizotinib treatment alone apparently increased MET protein levels and it has been reported that sustained activation of MET signaling leads to cardiac remodeling and heart failure [35,36]. These results prompted us to examine the effect of crizotinib on MET and the role of MET in crizotinib-induced cardiac complications. We found that crizotinib could increase the protein level of MET in both MCMs and CCC-HEH-2 cells (Figure 2E, F). However, the phosphorylation of MET at Tyr1234/1235, which is critical for its kinase activity, remained at a low level (Figure S3A) in both control and crizotinib-treated cardiomyocytes, indicating the limited kinase activity of MET in cardiomyocytes. Immunofluorescence assay further showed increased MET protein in crizotinib-treated cardiomyocytes (Figure 2G and Figure S3B) and hearts from crizotinib-treated mice (Figure 2H). In addition, we found that overexpression of MET caused upregulation of MET and c-PARP protein levels (Figure S3C) and increased apoptosis rate of cardiomyocytes (Figure S3D) as detected by Flow cytometry with PI and ANXA5 co-staining. In addition, overexpression of MET also led to reduced MMP (Figure S3E). These results suggested that increased MET protein contributes to crizotinib-induced cardiomyocyte death. Notably, savolitinib, another MET kinase inhibitor, could not increase MET or c-PARP protein level (Figure 2I and Figure S3F), suggesting that MET-dependent apoptosis might not be caused by the inhibition of MET kinase activity.

**Figure 3.**
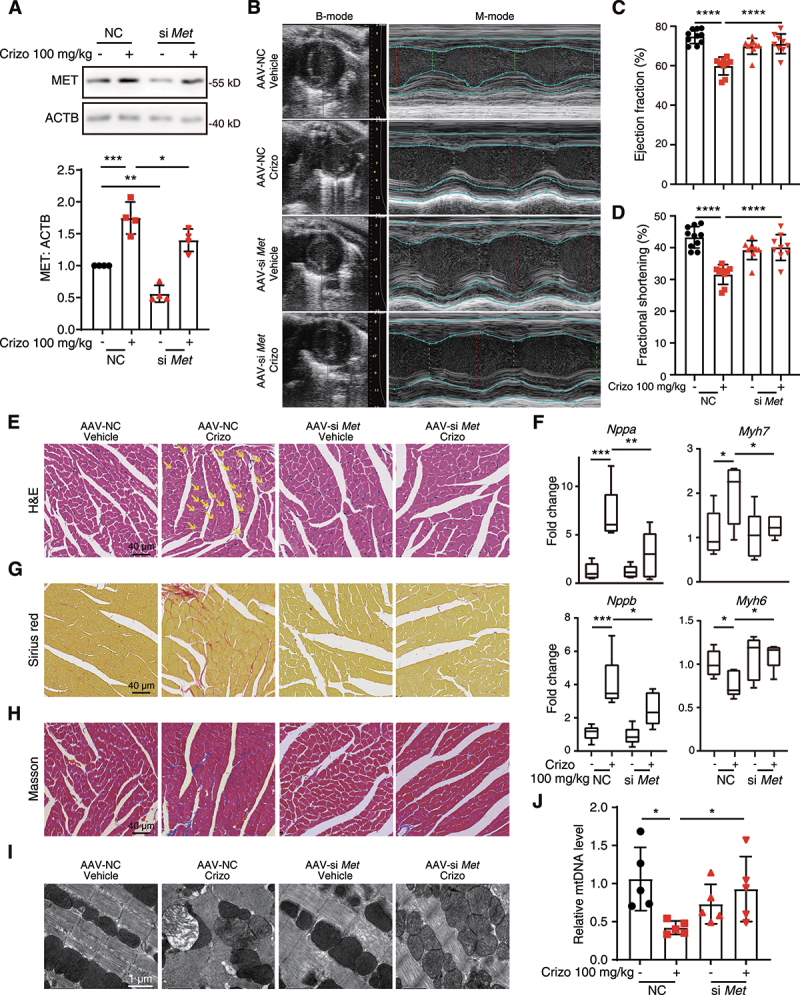
Silence of the *Met* gene through AAV9 attenuates crizotinib-induced cardiac complications *in vivo*. AAV9-*TNNT2*-si *Met* or AAV9-*TNNT2*-NC virus was injected into C57BL/6J mice (*n* ≥ 9) through the tail vein. Three weeks after injection, mice were then intragastrically administrated with vehicle or 100 mg/kg crizotinib for another 6 weeks. (A) Total protein was extracted from mice hearts (*n* = 4). Representative immunoblots and relative quantification of the MET were shown. ACTB was used as a loading control. (B-D) the heart function of mice was measured by echocardiography. (E) H&E staining. The yellow arrow indicated typical pathological changes. Scale bar: 40 μm. (F) the cardiac remodeling gene expression relative to *Actb* was shown. *n* = 5. (G and H) Representative images of cardiac sections stained by Sirius red or Masson from C57BL/6J mice. Scale bar: 40 μm. (I) TEM observation of the cardiac ventricles from mice (A) and representative images were shown. Scale bar: 1 μm. (J) the *mt-Atp6*: *Rpl13* ratio was measured as a relative mtDNA level. *n* = 5. Data were presented as mean ± SD. The *P* value was calculated by one-way ANOVA with Sidak’s test (A, C, D, F and J) multiple comparisons test. ****, *P* < 0.0001; ***, *P* < 0.001; **, *P* < 0.01; *, *P* < 0.05. Crizo: crizotinib.

Given the role of increased MET protein in cardiomyocyte death, we then further verified the relationship between MET protein and cardiac complications of crizotinib *in vivo*. We established a mouse model of cardiomyocyte-specific *Met* gene knockdown by injecting mice with adeno-associated virus 9 (AAV9) carrying the *TNNT2* promoter with siRNA targeting the *Met* gene (AAV-si *Met*) and we confirmed the knockdown efficiency by WB (Figure 3A) and qPCR (Figure S3G). As detected by echocardiography, the knockdown of the *Met* gene rescued crizotinib-reduced EF and FS (Figure 3B–D), suggesting the recovery of left ventricular dysfunction. Knockdown of the *Met* gene also abolished elevated serum CKMB levels (Figure S3H) and myocardial injury (Figure 3E) in crizotinib-treated mice as detected by H&E staining. The expression level of *Nppa*, *Nppb*, *Myh6* and *Myh7* genes in AAV9-si *Met*-injected mice treated with crizotinib kept unaltered (Figure 3F), which was consistent with reduced collagen deposition (Figure 3G, H). TEM results showed normal mitochondrial structure in ventricular tissues from AAV9-si *Met*-injected mice treated with crizotinib (Figure 3I). The reduced *mt-Atp6*: *Rpl13* ratio was also upregulated by *Met* knockdown (Figure 3J). Taken together, we demonstrated that crizotinib induces cardiac complications by promoting the aberrant accumulation of MET protein.

### Crizotinib inhibits the autophagic degradation of MET by perturbing autophagosome-lysosome fusion

**Figure 4.**
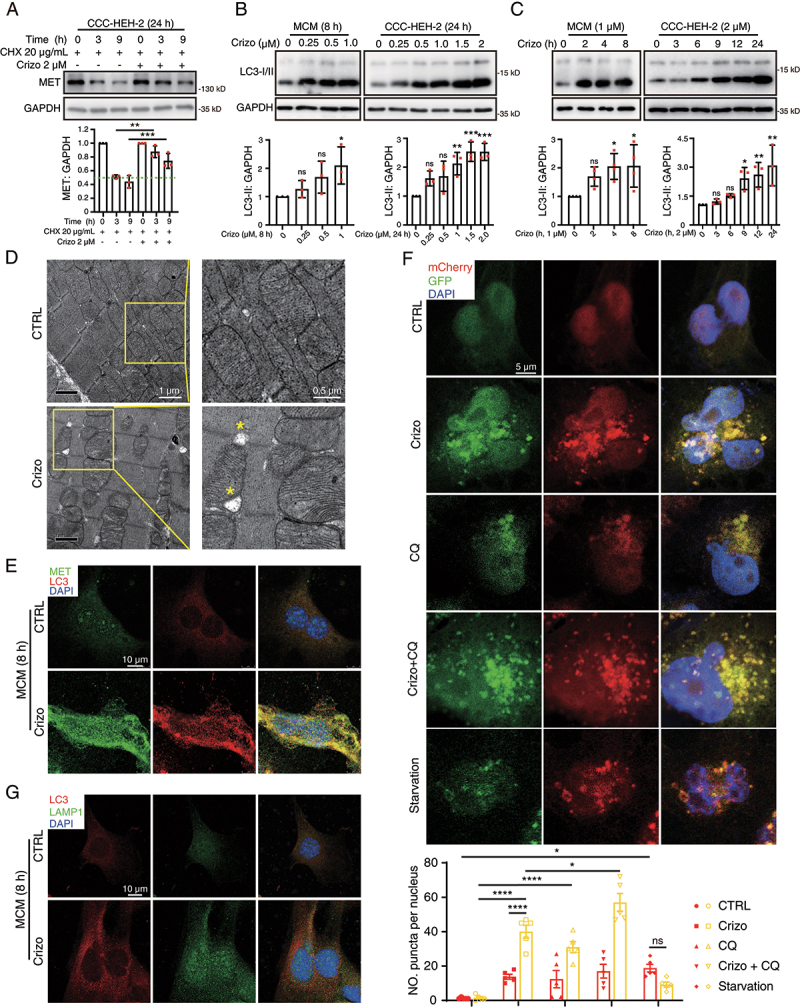
Crizotinib inhibits autophagosome-lysosome fusion in cardiomyocytes. (A) CCC-HEH-2 cells were treated with CHX (20 μg/mL) with or without crizotinib. Representative images (upper) and relative quantification (lower) were shown. GAPDH was used as a loading control. *n* = 3. (B, C) MCMs and CCC-HEH-2 cells were treated with crizotinib as indicated. Representative images (upper) and relative quantification (lower) were shown. GAPDH was used as a loading control. *n* = 3, 4. (D) TEM observation of the cardiac ventricles from mice treated with crizotinib and representative images were shown. Scale bar: 1 μm, 0.5 μm. (E) the co-localization of LC3 with MET in untreated or crizotinib-treated MCMs. The cells were stained for LC3 (red), MET (green) and DAPI (blue). Cells were treated with crizotinib for 8 h. Scale bar: 10 μm. (F) MCMs were infected with the mCherry-GFP-LC3 virus. 12 h after infection, cells were treated with crizotinib (2 μM), CQ (10 μM) and CQ plus crizotinib for 8 h or serum starvation stress for 2 h, respectively. Autophagic flux assays were performed with the confocal microscope. Scale bar: 5 μm. Quantification of the number of mCherry and GFP fluorescent puncta was beneath the images. *n* = 10 fields from more than three independent experiments per group. (G) the cells were treated with crizotinib for 8 h cells and then were stained for LC3 (red), LAMP1 (green) and DAPI (blue). Scale bar: 10 μm. Data were presented as mean ± SD. The *P* value was calculated by one-way ANOVA with Dunnett’s (B and C), Tukey’s (A and E) multiple comparisons tests or unpaired Student’s t test (F, within a group). ****, *P* < 0.0001; ***, *P* < 0.001; **, *P* < 0.01; *, *P* < 0.05; ns, no significance. CTRL: control; Crizo: crizotinib; LAMP1: lysosomal-associated membrane protein 1.

We next sought to clarify the mechanism for MET accumulation. qPCR analysis showed no significant change in the mRNA level of *MET* in crizotinib-treated CCC-HEH-2 cells (Figure S4A). We then applied cycloheximide (CHX), an inhibitor of protein synthesis, to detect the effect of crizotinib on the turnover of MET protein. Western blot results showed that crizotinib extended the half-life of MET (Figure 4A), suggesting that increased MET protein is caused by degradation inhibition. These findings promoted us to detect how crizotinib prevents the degradation of MET.

It has been reported that the autophagy-lysosome pathway contributes to the selective degradation of MET in cancer cells [37] and proteasome is vital for protein degradation. Therefore, we first applied MG132, a specific proteasome inhibitor, chloroquine (CQ), a lysosome inhibitor, and 3-MA, an autophagosome inhibitor, to examine the role of autophagy-lysosome and proteasome pathway in the degradation of MET protein in cardiomyocytes. Western blot results showed that MG132 could not prevent the degradation of MET (Figure S4B), but CQ and 3-MA could (Figure S4C, D), suggesting that lysosomes rather than proteasomes contribute to MET degradation. In addition, the silence of *ATG5* or *ATG7*, genes required for autophagosome formation, could inhibit the degradation of MET (Figure S4E). These data suggested that MET protein is degraded by the autophagy-lysosome pathway in cardiomyocytes and crizotinib might inhibit the autophagic degradation of MET. The degradation of a substrate protein by autophagy undergoes several steps, including autophagy induction, substrate recognition by autophagosome, autophagosome-lysosome fusion and final degradation. Therefore, we evaluated the effects of crizotinib on these steps to reveal how crizotinib prevents the degradation of MET. Increased MAP1LC3/LC3-II level is a marker for autophagy induction and LC3 is also used as the marker for autophagosome. We found that crizotinib treatment significantly increased LC3-II levels in both MCMs and CCC-HEH-2 cells (Figure 4B, C) and TEM analysis revealed accumulated autophagosomes in crizotinib-treated hearts (Figure 4D), suggesting that crizotinib could not inhibit autophagy induction.

Immunofluorescence assay showed that crizotinib promoted the accumulation and the overlay of MET and LC3 protein in MCMs (Figure 4E and Figure S4F), suggesting that MET could be engulfed by autophagosome upon crizotinib treatment, further indicating that MET protein is degraded in an autophagy-dependent manner. Thus, we further investigated whether crizotinib could affect the autophagosome-lysosome fusion by detecting autophagy flux via infecting CCC-HEH-2 and MCM cells with the Ad-mCherry-GFP-LC3 virus. We found that almost all the LC3 puncta in crizotinib-treated CCC-HEH-2 cells are with yellow fluorescence (autophagosomes) even after 24 h of treatment (Figure 4F and Figure S5A, B), suggesting the loss of green fluorescence by an acidic environment in lysosome was blocked by crizotinib, indicating the impaired autophagosome-lysosome fusion. As expected, amino acid deprivation enhanced autophagy flux, as shown by increased red puncta (autolysosomes) and treatment with lysosome inhibitor CQ showed similar yellow puncta as crizotinib (Figure 4F). Notably, co-treatment with both CQ and crizotinib further increased the number of yellow puncta (Figure 4F), indicating that crizotinib might impair autophagosome-lysosome fusion in a manner different from CQ, namely in a lysosome-independent mechanism. In addition, the accumulation of yellow puncta by crizotinib was further confirmed in MCMs (Figure S5C, D). Consistent with this, crizotinib treatment increased the puncta number of LAMP1 (lysosomal-associated membrane protein 1) and LC3, but could not promote the overlay of these two proteins (Figure 4G). Taken together, these results indicated that crizotinib impairs autophagosome processing and autophagic degradation by inhibiting autophagosome-lysosome fusion, which leads to the aberrant accumulation of MET protein.

### Crizotinib perturbs autophagosome-lysosome fusion by inhibiting the phosphorylation of PRKAA (Ser485/491)

**Figure 5.**
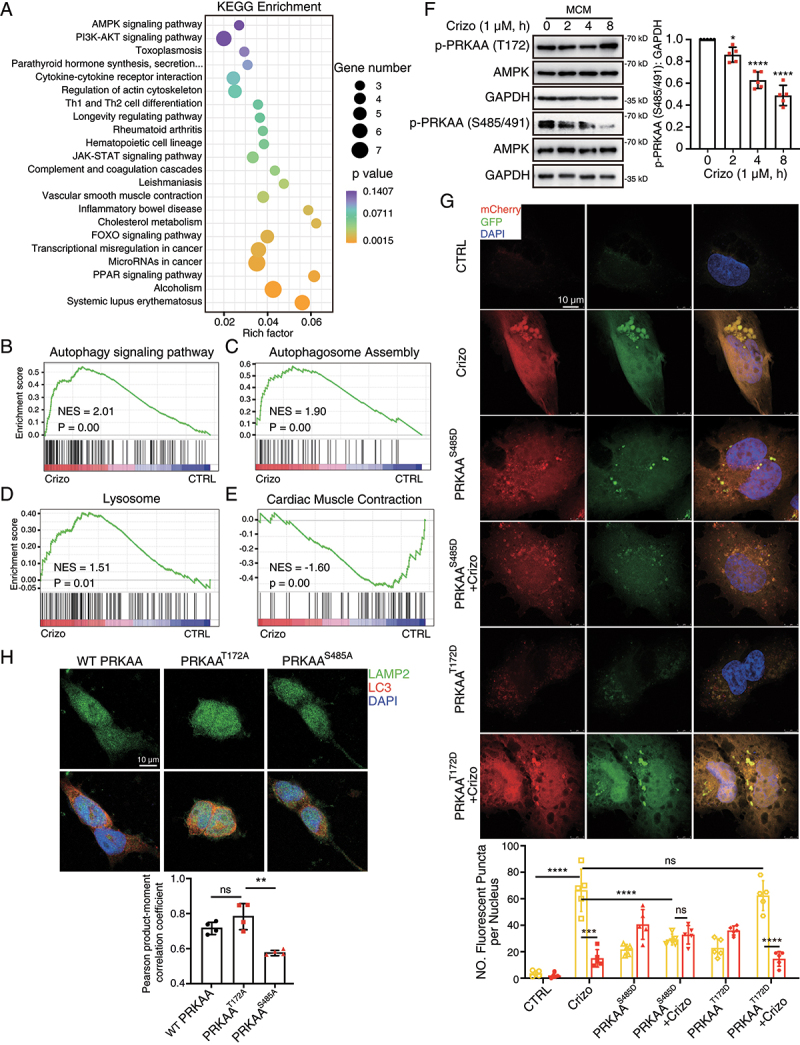
PRKAA (Ser485/491) phosphorylation regulates crizotinib-induced fusion disorders between autophagosomes and lysosomes. (A-E) CCC-HEH-2 cells were treated with 2 μM crizotinib for 12 h and subjected to RNA-Sequencing analysis. (A) KEGG pathway terms of differentially expressed genes between crizotinib-treated and the control cells. (B-E) GSEA on specific pathways as indicated. (F) MCMs were treated with crizotinib. GAPDH was used as a loading control. The quantification of p-PRKAA (S485/491) was shown on the right. *n* = 5. (G) CCC-HEH-2 cells were infected with the mCherry-GFP-LC3 virus. 12 h after infection, cells were transfected with PRKAA^S485D^ and PRKAA^T172D^ plasmid, respectively. Then 6 h after transfection, cells were treated with crizotinib (2 μM, 24 h). Autophagic flux assays were performed with the confocal microscope. Scale bar: 20 μm. Quantification of the number of mCherry and GFP fluorescent puncta was beneath the images. *n* = 5 fields per group. (H) Representative immunofluorescence images of 293T cells stained by LAMP2A and LC3 antibody. Scale bar: 50 μm. Pearson correlation coefficients were analyzed as LAMP2A and LC3 co-localization rates. *n* = 4 fields from more than three independent experiments per group. Data were presented as mean ± SD. The *P* value was calculated by one-way ANOVA with Dunnett’s (F), Tukey’s (G) or Sidak’s (H) multiple comparisons tests. ****, *P* < 0.001; ***, *P* < 0.001; **, *P* < 0.01; *, *P* < 0.05; ns, no significance. CTRL: control; Crizo: crizotinib.

We then investigated the mechanism for crizotinib-impaired autophagosome-lysosome fusion by performing RNA-Sequencing analysis on crizotinib-treated CCC-HEH-2 cells. We found that the expression of 328 genes was significantly upregulated (fold change ≥ 1, q ≤ 0.05), in crizotinib-treated cells compared with control, while the expression of 177 genes was downregulated (Figure S6A and Table S1). Kyoto Encyclopedia of Genes and Genomes (KEGG) pathway enrichment analysis showed enrichment of upregulated genes involved in AMPK signaling and phosphoinositide 3-kinase (PI3K) signaling (Figure 5A), which are closely related to cardiac energy metabolism homeostasis and autophagy activity. In addition, Gene Set Enrichment Analysis (GSEA) further showed that signaling pathways including Autophagy signaling, Autophagosomes assembly and Lysosome (Figure 5B–D) were more enriched in crizotinib-treated cells, which were consistent with the autophagy induction in cardiomyocytes caused by crizotinib. Furthermore, GSEA results showed that genes involved in Cardiac Muscle Contraction, Oxidative Phosphorylation, Mitochondrial Inner Membrane, Mitochondrial Respiratory Chain Complex I Assembly, Mitochondrial Electron Transport NADH to Ubiquinone, ATP Biosynthetic Process, Mitochondrial Large, Small Ribosomal Subunit and Translation were generally downregulated upon crizotinib treatment (Figure 5E and Figure S6B-I), which could explain the abnormal mitochondrial and decreased MMP that were observed in Figure 2.

AMPK plays key functions in regulating autophagosome-lysosome fusion [21,22], and is required for cardiac function and homeostasis. Therefore, we sought to investigate whether AMPK was involved in the cardiotoxic effect of crizotinib. We found that glucose deprivation, which could activate the phosphorylation of PRKAA/AMPKα (Ser485/491 and Thr172), apparently reduced MET and apoptotic proteins (Figure S7A), suggesting that PRKAA activation by glucose deprivation might protect cardiomyocytes from death. Therefore, we then detect the effects of crizotinib on the phosphorylation of PRKAA and found that crizotinib promoted the phosphorylation of PRKAA (Thr172) in MCMs (Figure 5F) and CCC-HEH-2 cells (Figure S7B, C), which was consistent with the autophagy induction. Consistently, we found that crizotinib promoted the phosphorylation of ACAC/ACC (acetyl-CoA carboxylase; Ser79) (Figure S7D), an PRKAA target, suggesting the activation of canonical AMPK signaling. Notably, the phosphorylation of PRKAA at Ser485/491 was inhibited by crizotinib (Figure 5F, and Figure S7B, C). It has been reported that cadmium could inhibit the phosphorylation of Ser485 and cadmium could impair autophagosome-lysosome fusion [24]. These findings prompted us to examine whether Ser485/491, which are conserved sites within PRKAA1 or PRKAA2, respectively, are the essential residues that maintain autophagosome-lysosome fusion.

As expected, the silence of the *PRKAA* gene prevented starvation-induced co-localization of LAMP2 (lysosomal-associated membrane protein 2) and LC3 (Figure S7E), the marker for lysosome and autophagosome respectively, confirming the importance of PRKAA in autophagosome-lysosome fusion in cardiomyocytes. We then constructed two constitutively phosphorylated PRKAA plasmids, wherein Thr172 or Ser485 residue was mutated to aspartic acid respectively, namely PRKAA^T172D^ and PRKAA^S485D^. We found that overexpression of PRKAA^S485D^ plasmid reduced the number of yellow puncta, increased the number of red puncta and reduced the puncta size in crizotinib-treated CCC-HEH-2 cells (Figure 5G and S7F), suggesting the recovery of autophagosome-lysosome fusion by PRKAA^S485D^. However, the PRKAA^T172D^ plasmid failed to do so (Figure 5G). In addition, we constructed two dominant negative mutant PRKAA plasmids, wherein Thr172 or Ser485 residue was mutated to alanine respectively, namely PRKAA^T172A^ and PRKAA^S485A^. We found that overexpression of WT or PRKAA^T172A^ plasmid could promote the co-localization of LAMP2 and LC3, while PRKAA^S485A^ showed much less effect without affecting the LAMP2 level (Figure 5H). Notably, overexpression of PRKAA^S485A^ plasmid showed slight cytotoxic effects on CCC-HEH-2 cells and overexpression of PRKAA^S485D^ plasmid promoted the survival of CCC-HEH-2 cells (Figure S7G), confirming that the phosphorylation of PRKAA (Ser485/491) is required for cardiomyocyte survival. In addition, PRKAA^S485A^ or PRKAA^S485D^ showed nearly no effects on the phosphorylation of PRKAA (Thr172) in CCC-HEH-2 cells (Figure S7H), suggesting that increased phosphorylation level of PRKAA (Thr172) is independent on the inhibition of Ser485/491 phosphorylation in crizotinib-treated cardiomyocytes. In summary, our data highlight the function of PRKAA (Ser485/491) phosphorylation in cardiomyocyte survival and autophagosome-lysosome fusion, which could reflect the activation of noncanonical AMPK signaling.

To investigate the mechanism for AMPK-mediated autophagosome-lysosome fusion in cardiomyocytes, we further analyzed the RNA-Sequencing data. As shown in Figure 5A and S8A, KEGG analysis and GSEA showed enrichment of genes involved in Forkhead box O (FOXO) signaling in crizotinib-treated cardiomyocytes. FOXO transcriptional factors, especially FOXO1, play important roles in the autophagy process and survival of cardiomyocytes by translocating to the nucleus and regulating the expression of genes involved in autophagosome-lysosome fusion [38,39]. Therefore, we sought to investigate the role of FOXO1 signaling in AMPK-mediated autophagosome-lysosome fusion in cardiomyocytes. Immunofluorescence assay results showed that crizotinib treatment prevented the nuclear localization of FOXO1 and immunoprecipitation assay revealed that the interaction between AMPK and FOXO1 was abolished by crizotinib (Figure S8B, C). We further found that PRKAA^S485D^ but not PRKAA^T172D^ could induce the nuclear localization of FOXO1 (Figure S8D). PRKAA^S485A^ could also inhibit the nuclear localization of FOXO1 (Figure S8D). Therefore, phosphorylation of PRKAA (Ser485/491) could maintain the nuclear localization of FOXO1, which is required for autophagosome-lysosome fusion, suggesting that PRKAA might regulate autophagosome-lysosome fusion in cardiomyocytes in a FOXO1-dependent manner.

We then explored the mechanism by which crizotinib prevents the PRKAA (Ser485/491) phosphorylation. The PI3K-AKT pathway is one of the downstream signaling pathways of ALK, the main target of crizotinib, and our RNA-Sequencing results revealed that genes related to the PI3K-AKT signaling pathway showed aberrant expression levels in crizotinib-treated cardiomyocytes (Figure 5A). Moreover, it has been reported that AKT could mediate the phosphorylation of PRKAA at Ser485/491 in skeletal muscle [40]. Therefore, we detect the role of AKT in the regulation of PRKAA (Ser485/491) phosphorylation. As expected, crizotinib or ceritinib, the ALK inhibitors, could reduce the phosphorylation of AKT (Ser473) (Figure S8E). In addition, silence the expression of *AKT1* or *AKT2* gene reduced the level of PRKAA (Ser485/491) phosphorylation (Figure S8F) in CCC-HEH-2 cells. These data suggested that AKT inhibition might contribute to crizotinib-reduced the level of PRKAA (Ser485/491) phosphorylation and PI3K-AKT signaling pathway could be the upstream of the phosphorylation of PRKAA (S485/491). Taken together, our data indicated that crizotinib blocks autophagosome-lysosome fusion in cardiomyocytes by inhibiting AKT-AMPK-FOXO1 signaling axis.

### Recovering PRKAA (Ser485/491) phosphorylation rescues crizotinib-induced cardiac complications

**Figure 6.**
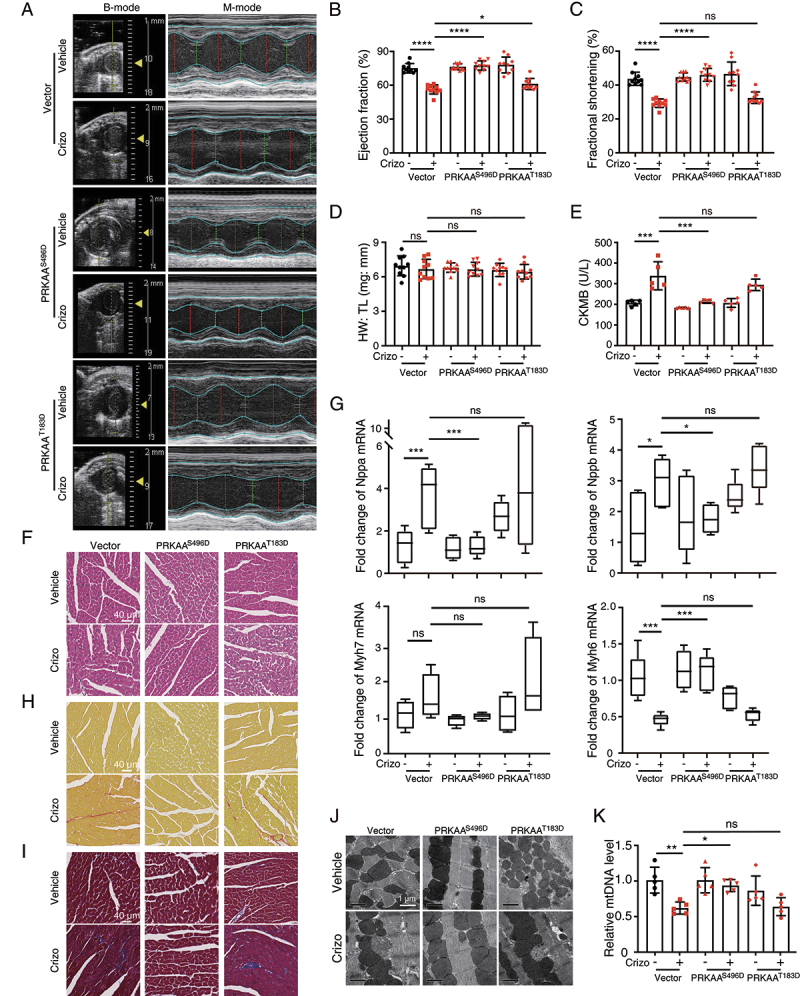
PRKAA^S496D^ overexpression attenuates crizotinib-induced cardiotoxicity. AAV9-*TNNT2*-vector, AAV9-*TNNT2*-PRKAA^S496D^ or AAV9-*TNNT2*-PRKAA^T183D^ virus was injected into C57BL/6J mice (*n* = 10). Three weeks after injection, mice were then intragastrically administrated with vehicle or 100 mg/kg crizotinib for another 6 weeks. (A-C) the left ventricular function of mice was measured by echocardiography. (D) heart weight to tibia length ratio (HW: TL). (E) serum was analyzed for CKMB level. *n* = 5. (F) H&E staining. Scale bar: 40 μm. (G) the gene expression levels relative to *Actb* in the indicated groups. *n* ≥ 4. (H, I) Representative Sirius red-stained (upper) and Masson-stained (lower) cardiac sections. Scale bar: 40 μm. (J) TEM observation of the left ventricle from C57BL/6J mice shown in A. (K) the *mt-Atp6*: *Rpl13* ratio was measured as a relative mtDNA level. *n* = 5. Data were presented as mean ± SD. The *P* value was calculated by one-way ANOVA with Sidak’s tests (B to D, F, G and K). ****, *P* < 0.001; ***, *P* < 0.001; **, *P* < 0.01; *, *P* < 0.05; ns, no significance. Crizo: crizotinib.

Based on our findings above, we speculated that recovering the phosphorylation of PRKAA (Ser485/491) could rescue the cardiac complications of crizotinib. Therefore, we cardiomyocyte-specifically overexpressed PRKAA^T183D^ (PRKAA Thr172 in humans was identified as Thr183 in mice) or PRKAA^S496D^ (PRKAA Ser485 in humans was identified as Ser496 in mice) in mice by injecting mice with AAV9 carrying the *TNNT2* promoter with PRKAA^T183D^ or PRKAA^S496D^. Echocardiography showed that overexpression of PRKAA^S496D^ alleviated the reduced left ventricular function caused by crizotinib (Figure 6A–C). Overexpression of PRKAA^S496D^ showed little effect on HW: TL ratio in crizotinib-treated mice (Figure 6D). Increased CKMB level (Figure 6E) and myocardial injury (Figure 6F) were prevented by PRKAA^S496D^ overexpression. In addition, the expression of *Nppa*, *Nppb*, *Myh6* and *Myh7* genes in hearts from AAV9-PRKAA^S496D^-injected mice treated with crizotinib reached the normal level (Figure 6G) and collagen deposition was barely observed (Figure 6H, I), suggesting the adverse cardiac remodeling caused by crizotinib was abolished by PRKAA^S496D^. Furthermore, PRKAA^S496D^ mitigated crizotinib-induced mitochondrial injury as detected by TEM (Figure 6J) and mitochondrial DNA level (Figure 6K). Notably, overexpression of PRKAA^T183D^ showed little effect on crizotinib-induced cardiac complications. Taken together, these results demonstrated that crizotinib causes cardiac complications by inhibiting PRKAA (Ser485/491) phosphorylation, which could be the potential therapeutic target for crizotinib-induced cardiac complications.

**Figure 7.**
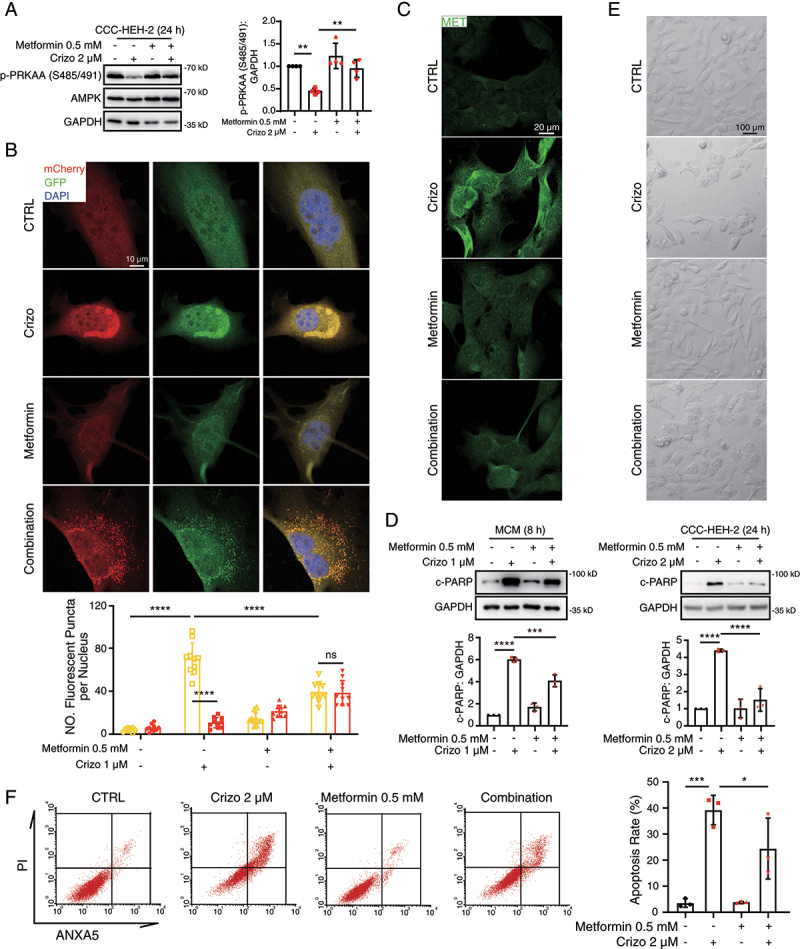
Metformin shows cardioprotective effects in crizotinib-induced cardiomyocyte death. (A) CCC-HEH-2 cells were treated with crizotinib with or without metformin. GAPDH was used as a loading control. Relative quantification was shown on the right. *n* = 4. (B) MCMs were injected with the mCherry-GFP-LC3 virus. 12 h after injection, cells were treated with crizotinib with or without metformin. Autophagic flux assays were performed with the confocal microscope. Scale bar: 10 μm. Quantification of the number of mCherry and GFP fluorescent puncta was beneath the images. *n* = 10 fields per group. (C) Representative images of MET staining on MCMs treated with crizotinib with or without metformin. Scale bar: 20 μm. (D) MCMs and CCC-HEH-2 cells were treated with crizotinib with or without metformin for 24 h. Total cell lysates were used to detect the expression levels of c-PARP. GAPDH was used as a loading control. Representative images (upper) and relative quantification (lower) were shown as in. *n* = 3. (E) CCC-HEH-2 cells were treated with crizotinib with or without metformin. Representative images of cell morphology were obtained by microscope. Scale bar: 100 μm. (F) CCC-HEH-2 cells were treated with crizotinib with or without metformin. And then cells were harvested and stained with PI and ANXA5, and apoptosis rates were detected by flow cytometry. *n* = 3. Representative images were shown on the left and a statistical histogram was presented on the right. Data were presented as mean ± SD. The *P* value was calculated by one-way ANOVA with Sidak’s test (A, D and F) or Student’s t test (B, within a group). ****, *P* < 0.001; ***, *P* < 0.001; **, *P* < 0.01; *, *P* < 0.05; ns, no significance. CTRL: control; Crizo: crizotinib.

Considering that metformin, a well-known inducer of PRKAA phosphorylation, shows cardiac protection in heart diseases [41], we then examined the therapeutic potential of metformin on crizotinib-induced impaired PRKAA phosphorylation, autophagy process, MET accumulation and cardiac complications. As shown in Figure 7A, the addition of metformin reversed the decline in PRKAA (Ser485/491) phosphorylation. Autophagy flux assay revealed that metformin abrogated the accumulated yellow puncta (Figure 7B) and reduce the increased puncta size caused by crizotinib (Figure S9A), suggesting that metformin could rescue the crizotinib-impaired autophagosome-lysosome fusion in cardiomyocytes. Consistent with this, we found that metformin impeded the increased MET protein level and cardiomyocyte apoptosis (Figure 7C–F) caused by crizotinib, indicating the protective effects of metformin in crizotinib-induced cardiomyocyte injury. In addition, we found that overexpression of PRKAA^S485A^ plasmid reversed the protective effects of metformin on cardiomyocyte apoptosis (Figure S9B-D), further suggesting that metformin mitigates crizotinib-induced cardiomyocyte death by recovering PRKAA (Ser485/491) phosphorylation.

**Figure 8.**
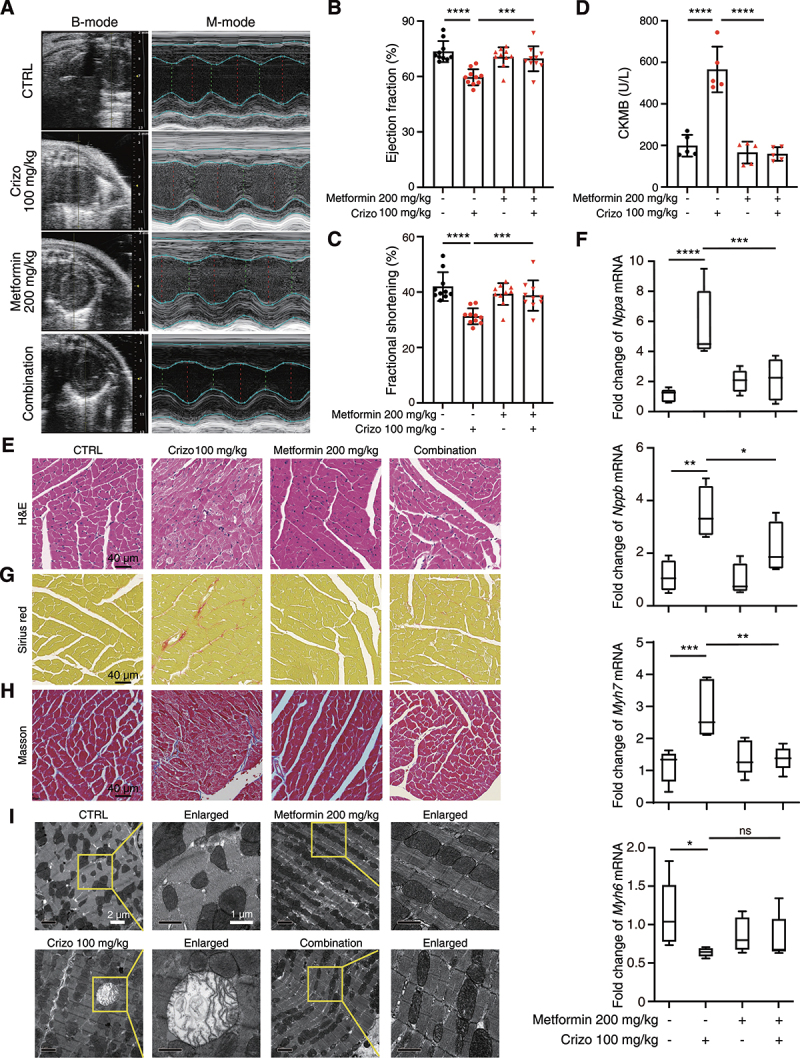
Metformin attenuates crizotinib-induced cardiac complications *in vivo*. C57BL/6J mice (*n* = 10) were treated with vehicle, crizotinib (100 mg/kg), metformin (200 mg/kg) or metformin plus crizotinib for 6 weeks by means of intragastric administration. (A-C) the heart function of mice was measured by echocardiography. (D) serum was analyzed for CKMB level. *n* = 5. (E) H&E staining. Scale bar: 40 μm. (F) the cardiac remodeling gene expression relative to *Actb* in the indicated groups was shown. *n* ≥ 4. (G, H) Representative Sirius red and Masson-stained cardiac sections. Scale bar: 40 μm. (I) TEM observation of the left ventricle from C57BL/6J mice showed in (A). Representative images of the mitochondrial structure were shown on the left and enlarged views were presented on the right. Data were presented as mean ± SD. *n* = 3. The *P* value was calculated by one-way ANOVA with Sidak’s test (B to D and F). ****, *P* < 0.0001; ***, *P* < 0.001; **, *P* < 0.01; *, *P* < 0.05; ns, no significance. CTRL: control; Crizo: crizotinib.

We next investigated this therapeutic potential of metformin *in vivo* by treating mice with crizotinib and, or metformin. The dose for metformin is about half of the clinically recommended daily dose. Echocardiography results showed that the combined application of metformin and crizotinib reversed crizotinib-reduced EF and FS (Figure 8A–C), indicating the protective effects of metformin on left ventricular function. Metformin also mitigated the elevated level of serum CKMB (Figure 8D), and myocardial injury (Figure 8E) caused by crizotinib. In addition, abnormal expression of pathological cardiac remodeling-related genes by crizotinib was abrogated by metformin (Figure 8F) and there was less collagen deposition in heart sections from mice treated with both crizotinib and metformin (Figure 8G, H). Furthermore, few mitochondria with the damaged structure were observed in ventricular tissues from mice treated with both crizotinib and metformin (Figure 8I). Taken together, our results demonstrated that the combined application of crizotinib and metformin rescued the cardiotoxicity, myocardial injury and adverse remodeling that were caused by crizotinib, suggesting that recovering PRKAA (Ser485/491) phosphorylation by metformin could be a potential preventive and therapeutic approach for cardiac complications caused by ALK inhibitors in clinic.

## Discussion

Several adverse effects, including cardiotoxicity, cutaneous toxicity, hepatotoxicity and pulmonary toxicity, have been reported in patients treated with crizotinib, which threatens the survival of patients and limits the clinical application of crizotinib. To solve this problem, we focused on the effects of crizotinib on normal cells and have previously reported that crizotinib showed cytotoxic effects on hepatocytes [42] and keratinocytes [43]. In addition, we further found that crizotinib could cause the death of cardiomyocytes in an autophagy-dependent manner. Considering the importance of cardiomyocytes in the heart, we mainly focused on the cytotoxic effects of crizotinib on cardiomyocytes and the involvement of the death of other cells including vascular endothelial cells and smooth muscle cells in cardiotoxicity needs to be further investigated. Moreover, we observed that metformin showed protective effects on the whole body of the mice, suggesting that the toxic mechanism that we proposed could also explain the other adverse effects caused by crizotinib.

It has been observed that several heart diseases are accompanied by reduced autophagy activity [5–7,44]. To investigate the role of autophagy, mice with deletion of genes related to autophagy were applied and several studies revealed that deletion of *Atg5* or *Atg7*, genes required for autophagosome formation, causes cardiac hypertrophy and dysfunction, and reduced the survival of mice [9,10]. In addition, the global deletion of *Lamp2*, a gene required for autophagosome-lysosome fusion [45], leads to defective mitochondrial and contractility in cardiomyocytes derived from induced pluripotent stem cells [46], and causes accumulation of autophagic vacuoles, reduced heart contractility in mice [47] and patients [48]. IR injury could also cause cardiomyocyte death by impairing autophagosome-lysosome fusion [12,49]. These reports showed that inhibition of either autophagy induction or autophagosome-lysosome fusion alone is sufficient to cause heart diseases, indicating persistent autophagy activity is essential for heart function and homeostasis. In this study, our data proved that crizotinib-impaired autophagosome-lysosome fusion contributed to mitochondrial injury and cardiomyocyte death. For the first time, we put forward the novel role of impaired autophagosome-lysosome fusion in drug-induced cardiac complications, which shed new light on the study of drug toxicology.

Impaired autophagosome-lysosome fusion causes mitochondrial permeabilization and cardiomyocyte death in IR injury [12]. Consistent with this finding, we found that crizotinib decreased MMP and disorganized mitochondrial structure in cardiomyocytes. Interestingly, we identified that impeded degradation of MET protein by crizotinib caused aberrant accumulated MET, which contributed to cardiac dysfunction, confirming the detrimental role of MET in the heart. Our data revealed MET as a specific autophagy substrate in cardiomyocytes, whose expression and kinase activity keep at a barely detectable level by autophagy-dependent degradation to maintain cell survival. Indeed, it has been reported that MET protein is substantially expressed in embryonic and neonatal cardiomyocytes and downregulated with ages [50]. Although one study showed that MET signaling exhibits a potential protective effect on cardiomyocytes in myocardial infarction [51], several studies demonstrated that cardiac-specific overexpression of constitutively activated MET causes cardiac hypertrophy, HF and death [35,36,52], suggesting the lethal effect of MET on postnatal hearts. Our data indicated that MET might promote cardiac injury by promoting mitochondrial injury via inhibiting the transcription of the network of genes involved in mitochondria, which preliminarily clarifies the possible mechanism for MET-mediated lethal effects on hearts. However, the detailed mechanism by which MET regulates the target genes` transcription remains to be elucidated in future study. Notably, the silence of *MET* gene expression or recovering MET degradation could prevent cardiomyocyte death, while the MET kinase inhibitors showed various effects on cardiomyocyte survival. These findings suggested that accumulated MET protein might promote cardiomyocyte death and cardiac dysfunction in a kinase-independent manner, which deserves further investigation. More importantly, targeting MET kinase activity directly might not be applicable to crizotinib-induced cardiotoxicity.

We have previously reported that maladaptive excessive autophagy contributes to sunitinib-induced cardiomyocyte death by degrading key substrate protein [53]. In this study, we showed that inadequate autophagy activity could also lead to cardiomyocyte death and cardiotoxicity. These findings suggested that fine-tuning of autophagy activity is required for cardiac function and homeostasis, and targeting aberrant autophagy activity could be a feasible strategy against autophagy-associated cardiac diseases, which requires the understanding of the precise regulatory mechanism for autophagy processes.

AMPK has been demonstrated as one of the most important regulators of autophagosome formation [20] and autophagosome-lysosome fusion [21,22]. In addition, a protective role of AMPK in heart diseases has been confirmed as deletion of the *Prkaa2* gene aggravates ischemic cardiac damage [14,15], cardiac hypertrophy [18] and HF by regulating mitophagy and mitochondrial homeostasis [17]. Taken together, these findings indicated that AMPK might maintain and protect cardiac function by orchestrating autophagy-mediated mitochondrial homeostasis, which was consistent with our findings that PRKAA inhibition causes reduced autophagy activity and cardiac injury. Notably, literature reported that Thr172 residue within PRKAA is phosphorylated, which reflects the activation of AMPK, in response to various cardiac pathological stress [54,55]. However, it is still not well understood why the activation of AMPK could not maintain persistent autophagy activity and heart function. Indeed, there are several other threonine or serine residues within PRKAA. Some studies suggested that phosphorylation at Ser485/491 showed inhibitory effects on AMPK activation. In the skeletal muscle, phosphorylation of PRKAA (Ser485/491) causes the degradation and inactivation of AMPK [40]. However, our data revealed that Ser485 within PRKAA1 or Ser491 within PRKAA2 is required for autophagosome-lysosome fusion, AMPK protein level kept unaltered when Ser485/491 phosphorylation were decreased and overexpression of PRKAA^S485A^ alone could cause cardiomyocyte death, pointing out that phosphorylation of PRKAA (Ser485/491) is required for noncanonical AMPK activation and cell survival, at least in cardiomyocytes, which deepens our understanding of the regulatory mechanisms for autophagy processes and AMPK activity. In addition, the cause for this conflict conclusion might come from the different physiological characteristics and transcription landscapes between cardiac and skeletal muscle.

The regulation mechanism for PRKAA (Ser485/491) phosphorylation is much less known than that for PRKAA (Thr172) phosphorylation. Our results revealed that both AKT1 and AKT2 could regulate the phosphorylation of PRKAA (Ser485/491) in cardiomyocytes, further confirming the findings in 2021 that AKT could mediate the phosphorylation of PRKAA (Ser485/491) in skeletal muscle [40]. PI3K-AKT pathway, one of the downstream signaling pathways of ALK [56] and could be inhibited by crizotinib, might reduce autophagosome-lysosome fusion in macrophages [57]. Considering the role of PRKAA (Ser485/491) phosphorylation in autophagosome-lysosome fusion, we speculated that AKT-AMPK signaling inhibition caused by crizotinib contributes to the defects of autophagosome-lysosome fusion in cardiomyocytes. Notably, PI3K-AKT signaling is closely related to tumor growth and metastasis. Therefore, recovering the phosphorylation of PRKAA (Ser485/491) without affecting the inhibitory effects on PI3K-AKT could be an applicable strategy for crizotinib-induced cardiac complications.

Metformin, pharmacological activators of AMPK by increasing the phosphorylation of PRKAA (Thr172), have been demonstrated to be able to prevent the progression of HF [41] and attenuated trastuzumab-induced cardiotoxicity [58]. However, the effects of metformin on the phosphorylation of PRKAA (Ser485/491) are unknown. In this study, we found that metformin apparently recovers crizotinib-decreased phosphorylation of PRKAA (Ser485/491) in cardiomyocytes. We further demonstrated that metformin could rescue cardiomyocyte death and cardiotoxicity caused by crizotinib via recovering the phosphorylation of PRKAA (Ser485/491). These findings confirmed the feasibility of metformin as a candidate drug for the treatment against the cardiotoxicity of crizotinib. In recent years, a number of retrospective and preclinical studies have also proposed the feasibility of metformin as an effective adjuvant therapy for tumor [59,60] and clinical studies showed that metformin significantly reduces lung cancer mortality [61]. These results further support that metformin in combination with crizotinib could be a safe and effective therapeutic strategy for lung cancer, which needs to be further investigated in clinic.

**Figure 9.**
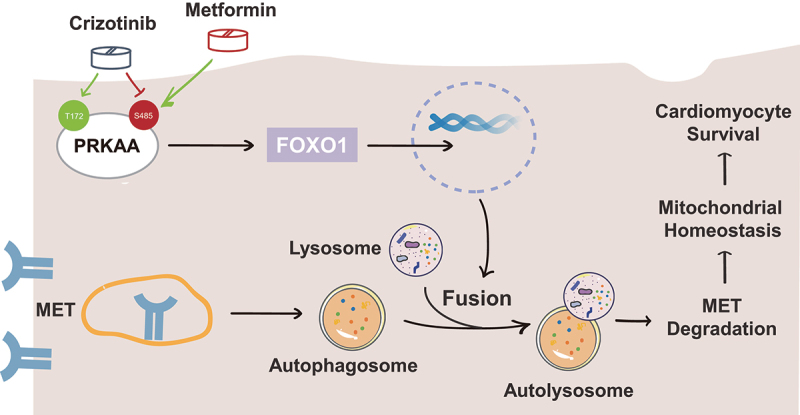
Schematic diagram illustrating the inadequate autophagy-mediated pathological process of crizotinib-induced cardiotoxicity and the application of metformin in preventing cardiotoxicity in patients treated with crizotinib.

Taken together, we elucidated the pathological process of crizotinib-induced cardiotoxicity (Figure 9), wherein inadequate autophagy activity leads to mitochondrial injury and cardiomyocyte death via the aberrant accumulation of MET protein. Our data showed that reduced phosphorylation level of PRKAA at Ser485/491 contributes to defective autophagosome-lysosome fusion and autophagosome maturation, which blocks autophagy activity, and recovering PRKAA (Ser485/491) phosphorylation-mediated autophagy flux by metformin could be a feasible and novel strategy to prevent the cardiotoxicity in patients with treatment of crizotinib.

## Materials and methods

### Animal study

All animal-related experimental procedures and methodologies were approved by the Center for Drug Safety Evaluation and Research of Zhejiang University and performed according to the Institutional Animal Care and Use Committee (IACUC) protocol of Zhejiang University (IACUC-s21–008). The C57BL/6J mice were purchased from Zhejiang Vital River Laboratory Animal Technology Co., Ltd. (Jiaxing, China) and housed in barrier facilities with a 12 h light-dark cycle and free food and water. The mice were housed for 1 week to adapt to the new environment before drug treatment or virus injection. Before tissue or blood collection, the mice were anesthetized with 2% isoflurane and then sacrificed via cervical dislocation.

Crizotinib (Meilunbio®, MB2025) and metformin (Sigma-Aldrich, M8699) were dissolved in cyclodextrin (Aladdin, H108813) to form a stock solution. The mice (7–9 weeks old) were treated with vehicle, 100 mg/kg crizotinib, 200 mg/kg metformin daily or the combination through intragastric administration for 42 days.

### Cardiomyocyte-specific silencing of *met* in mice

The AAV9 vectors carrying the *TNNT2* promoter and *Mir30*-siRNA were constructed and packaged by Tsingke Biotechnology (Hangzhou, China), namely AAV9-*TNNT2-Mir30*-si *Met* or AAV9-*TNNT2*-NC. The virus was purified and titers of the AAV vectors (viral genomes/mL) were measured by qPCR. The AAV9-*TNNT2*-NC (1.8 × 10^12^ vg/mL) and AAV9-*TNNT2-Mir30*-si *Met* (5 × 10^12^ vg/mL) were then injected into C57BL/6J mice (6 weeks old) through the tail vein (2.5 × 10^11^ vg per mouse). Three weeks later, mice with cardiomyocyte-specific knockdown of *Met* were treated with 100 mg/kg crizotinib for 42 days. Then, echocardiography was performed, CKMB levels were tested, and hearts were harvested for weight measurement and histological analysis. The siRNA sequences were as follows:

NC: 5’- TTCTCCGAACGTGTCACGTAA −3’;

si *Met*: 5’- CGGGATTCTTTCCAAACACTT −3’.

### Echocardiography

Cardiac function was evaluated in mice with 1% isoflurane (RWD Lifescience, R510–22) using a Vevo3100 Imaging System (Fujifilm Visual Sonics, Vevo3100). VisualSonics software was used to analyze the left ventricular internal diameter in diastole and systole and the end-diastolic volume or end-systolic volume. The left ventricular EF was calculated according to the following formula: EF (%) = [(end-diastolic volume – end-systolic volume)/end-diastolic volume] × 100. The left ventricular Fractional Shortening (FS) was calculated according to the following formula: FS (%) = [(left ventricular internal diameter in diastole – systole)/left ventricular internal diameter in diastole] × 100.

### Transmission electron microscopy analysis

Left ventricular tissues were collected from indicated mice. Tissue blocks of the left ventricle were cut out with a surgical instrument (far away from the apes of the heart to ensure consistent alignment of muscle filaments) and fixed in 1 mL fresh 2.5% glutaraldehyde (Scientific Phygene, PH9003) solution at room temperature for 2 h and stored at 4°C overnight. Tissue blocks were fixed with 1% osmic acid for 1 h and stained with 2% uranium for 0.5 h. After being dehydrated and embedded, the samples were sectioned with an ultrathin slicer. A transmission electron microscope (Thermo Fisher Scientific, TECNAI 10) was used to collect images.

### Biochemical analysis

Biochemical analysis was performed according to a previous study. After drug treatment, serum was obtained after centrifugation (2000 g for 10 min at 4°C) of the blood. The level of serum CKMB and LDH was observed with a fully automatic biochemical detection machine (Roche Diagnostics GmbH, Cobas c 311) with specific detection kits (Roche Diagnostics GmbH, 07190808190) according to the manufacturer’s instructions.

### Histological analysis

Heart samples were obtained from mice and fixed in 10% phosphate-buffered (composition: 137 mM NaCl, 2.7 mM KCl, 10 mM Na_2_HPO_4_, 1.76 mM K_2_HPO_4_, pH 7.4) formalin (Sigma-Aldrich, F8775), embedded in paraffin, and sectioned at 5-μm intervals. After de-waxing and rehydration, the sections were stained with hematoxylin and eosin, Sirius red, Masson’s trichrome or wheat germ agglutinin (Alexa Fluor^TM^ 488 Conjugate, 5 μg/mL; Thermo Scientific, W11261) for analysis.

### Cell culture and drug treatment

The human embryonic cardiac tissue-derived cell line CCC-HEH-2 (RRID: CVCL_VU29) was obtained from the National Infrastructure of Cell Line Resource of China. The cells were cultured in Dulbecco’s modified Eagle’s medium (DMEM; Gibco, 10569010) supplemented with 10% fetal bovine serum (FBS; HyClone, SV30160.03), 100 U/mL penicillin and 100 μg/mL streptomycin (Gibco, 10378016) in a humidified atmosphere with 5% CO_2_ at 37°C. All cell lines routinely tested negative for mycoplasma contamination.

The cells were treated with 1 or 2 μM crizotinib for the indicated time points or 0.25, 0.5, 1or 2 μM crizotinib for 24 h for cell survival analysis and western blots. In specific samples, 10 μM CQ (Sigma-Aldrich, C6628), 20 μg/mL CHX (MedChemExpress, HY-12,320) or 0.5 mM metformin was used.

### Isolation of adult mouse cardiomyocytes (MCMs)

Ventricular tissues were removed from adult mice and then modified Langendorff perfusion devices were employed. Then, Type II collagenase (Gibco, 17018029) was used to separate myocytes from the isolated ventricular tissues. Cardiomyocytes were separated from fibroblasts by differential plating and were cultured in media containing DMEM, 10% FBS, 100 μM bromodeoxyuridine, penicillin-streptomycin and L-glutamine.

### RNA-Sequencing analysis

CCC-HEH-2 cells were treated with or without crizotinib for 12 h, and total RNA was harvested with TRIzol reagent. After purification and quantification, the mRNA was reverse transcribed to 1 μg cDNA by SuperScript™ II Reverse Transcriptase (Invitrogen, 1896649). The cDNA was used for the construction of sequencing libraries using TruSeq RNA Sample Prep Kit (Illumina, FC-122-1001). The average insert size for the final cDNA library was 300 ± 50 bp. At last, the cDNA was sequenced using an Illumina Novaseq™ 6000 (LC-BIO Technologies [Hangzhou] Co., Ltd., Hangzhou, China) and the 2 × 150 bp paired-end run according to the manufacturer’s instructions. Kyoko Encyclopedia of Genes and Genomes (KEGG) and Gene Set Enrichment Analysis (GSEA) were performed on OmicStudio tools (https://www.omicstudio.cn/tool). RNA-sequencing data have been deposited at Gene Expression Omnibus under accession numbers GSE210150.

### Autophagy flux assay

Cells were seeded in Nunc™ Lab-Tek™ II Chamber Slide™ (Thermo Fisher Scientific, 154534) and infected with an adenovirus encoding the tandem fluorescent probe mCherry-GFP-LC3 and then treated as indicated in figure legends. Cells were fixed with 4% paraformaldehyde (Sigma-Aldrich, P6148) in phosphate-buffered saline (PBS; Gibco, 10010,023) for 20 min at room temperature. After washing with PBS, the cells were permeabilized with ice-cold 0.1% Triton X-100 (BioFROXX, 1139ML100) in PBS for 10 min and stained with DAPI (Dojindo, D212) for 5 min. Then cells were mounted for analysis by confocal microscope (Leica, TCS SP8,). Cells with appropriate mCherry-GFP-LC3 fluorescence were chosen and quantified by the Analyze Particles plugin in ImageJ as previously reported [53].

### Western blot analysis

After treatment, protein lysates were obtained from cells or tissues with IP buffer which contains 1% NP-40 (Beyotime, ST366), 0.3% Triton X-100, 0.25% leupeptin (Amresco, J580), 0.1% PMSF (Amresco, P8340) and 0.1% NaVO_3_ (Sigma-Aldrich, 450,243). Protein concentration was determined by BCA assay. Protein lysates (10–20 μg per sample) with loading buffer were separated on 8, 10 or 12% SDS-polyacrylamide gels and transferred to PVDF membranes (Sigma Millipore, IPVH00010) later. Membranes were blocked with 5% skim milk in 1× TPBS and then incubated with the antibodies listed below. Primary antibodies were diluted to (1:500–5000). After primary antibody incubation at 4°C overnight, membranes were incubated with secondary antibodies and signal detection followed the suppliers’ protocol using western Lightning Plus-ECL Enhanced Chemiluminescence Substrate (New Cell & Molecular Biotech, P2300). Quantitative image analysis was performed using Quantity One software according to the manufacturer’s protocol.

The following primary antibodies were used: anti-c-PARP (Rodent sample, Cell Signaling Technology, 94885S), anti-c-PARP (HuaBio Technology, ET1608–10), anti-HSPD1 (Santa Cruz Biotechnology, sc -59,567), anti-TOMM20 (Santa Cruz Biotechnology, sc -17,764), anti-LC3 (Cell Signaling Technology, 2775S), anti-MET (Cell Signaling Technology, 8198S), anti-MET (Rodent sample, Abcam, ab51067), anti-ACTB (Diagbio Technology, db7283), anti-PRKAA/AMPK (Cell Signaling Technology, 5832S), anti-p-PRKAA/AMPK (S485/491; Cell Signaling Technology, 4185S), anti-p-PRKAA/AMPK (T172; Cell Signaling Technology, 2535S), anti-FOXO1 (HuaBio Technology, ET1608–25), anti-GAPDH (Diag Technology, db106), anti-γ-H2AX (Cell Signaling Technology, 9718S), anti-BAX (Abclonal Technology, A19684), anti-CYCS (HuaBio Technology, ET1610–60), anti-p-MET-1234/1235 (Cell Signaling Technology, 3077S), anti-ATG5 (HuaBio Technology, ET1611–38), anti-ATG7 (Cell Signaling Technology, 8558S), anti-p-ACAC/ACC-S79 (Cell Signaling Technology, 11818T), anti-ACAC/ACC (ABclonal Technology, A15606), anti-AKT1 (Cell Signaling Technology, 2938S) and anti-AKT2 (Cell Signaling Technology, 3063S).

The following secondary antibodies were used: anti-mouse (Hangzhou Fude Biological Technology, FDM007) and anti-rabbit (Hangzhou Fude Biological Technology, FDR007).

### Immunofluorescence assay

For *in vitro* assay, cells were seeded on poly-D-lysine-coated coverslips. After treatment, cells were fixed with 4% paraformaldehyde in PBS for 20 min at room temperature. The cells were then permeabilized by 1% Triton X-100 in PBS for cells for 10 min at 4°C and blocked with 4% bovine serum albumin (Sigma-Aldrich, B2064) in PBS for 30 min.

For *in vivo* observation, heart tissues were collected and fixed with 4% paraformaldehyde overnight. Then the tissues were dehydrated with 30% sucrose buffer (Sinopharm Chemical Reagent, 10021,418) and embedded in Optimal Cutting Temperature (Sakura Finetek, 4583) to make frozen sections (10 μm). The sections were washed 3 times with 1× PBS and blocked with 1% bovine serum albumin in PBS with 0.1% Tween-20 (Aladdin, T104863) for 30 min.

Cells or sections were incubated with primary antibodies overnight, stained with secondary antibodies for 2 h and DAPI for 5 min and mounted for fluorescence microscope (Leica, TCS SP8).

The following primary antibodies were used: anti-MET (Cell Signaling Technology, 8198S), anti-MET (Rodent sample; Abcam, ab51067), anti-LC3 (Santa Cruz Biotechnology, sc -376,404), anti-LAMP1 (Santa Cruz Biotechnology, sc-5570), anti-LAMP2 (Santa Cruz Biotechnology, sc-5571), anti-γ-H2AX (Cell Signaling Technology, 9718S), anti-FOXO1 (HuaBio Technology, ET1608–25).

The following secondary antibodies were used: donkey anti-mouse (Invitrogen, A10037, A-21202) and donkey anti-rabbit (Invitrogen, A10042, A-21206).

### Cell survival assay

The cell survival assay was assessed by an SRB (Sigma-Aldrich, S1402) colorimetric assay. The absorbance at 515 nm was measured with a Multiscan Spectrum (Thermo Fisher Scientific, Marietta; USA). The cell survival fraction was calculated for each well as follows: the absorbance of treated cells/the absorbance of control cells × 100%.

### Cell transfection

Cells were seeded into 6-well plates at 8 × 10^4^ per well and grown to 50–60% confluence. si-jetPRIME (Polyplus-transfection; Illkirch, 114–15) was used according to the manufacturer’s recommendations. siRNA oligonucleotides were transfected at a final concentration of 12 nM. The transfection solution was changed to fresh medium plus 10% FBS for further study 6 h after transfection.

The following oligonucleotides were provided by GenePharma (Shanghai, China) as siRNAs targeting the indicated genes:

Negative Control sense: 5’-UUCUCCGAACGUGUCACGU-3’

si *MET* sense: 5’-GCCCAACUACAGAAAUGGU-3’

si *AKT1* sense: 5’- CGCGUGACCAUGAACGAGUUU-3’

si *AKT2* sense: 5’- CGGCUCCUUCAUUGGGUACAA −3’

### Quantitative real-time PCR (qPCR)

The detailed procedures were performed according to a previous study [53]. After treatment, the cells were harvested with TRIzol (Invitrogen, 15596,026) reagent. Equal amounts of RNA were reverse transcribed into complementary DNA and the genomic DNA was removed with TransScript® One-Step gDNA Removal and cDNA Synthesis SuperMix (TransGene Biotech, AT311–03). qPCR was performed on a 7500 Fast System using iTaq Universal SYBR Green Supermix (Bio-Rad Laboratories, 172–5124) and a variable volume of water to generate a volume of 20 μL. The samples underwent two-step amplification with an initial step at 95°C (3 min), followed by 39 cycles of 95°C (3 s) and 60°C (31 s). The melting curve was analyzed. Fold changes in the expression of each gene were calculated by the comparative threshold cycle (Ct) method using the formula 2^−(ΔΔCt)^.

The primer sequences were as follows:

*Nppa* forward: GAGAGAAAGAAACCAGAGTG

*Nppa* reverse: GTCTAGCAGGTTCTTGAAATC

*Nppb* forward: AATTCAAGATGCAGAAGCTG

*Nppb* reverse: GAATTTTGAGGTCTCTGCTG

*Myh6* forward: AATCCTAATGCAAACAAGGG

*Myh6* reverse: CAGAAGGTAGGTCTCTATGTC

*Myh7* forward: TTGGGAAATTCATCCGAATC

*Myh7* reverse: CCAGAAGGTAGGTCTCTATG

*mt-Atp6* forward: TCCTAGGCCTTTTACCACATACA

*mt-Atp6* reverse: TTTGTGTCGGAAGCCTGTAA

*Actb* forward: GTGACGTTGACATCCGTAAAGA

*Actb* reverse: GCCGGACTCATCGTACTCC

*Rpl13* forward: CCTGCTGCTCTCAAGGTTGT

*Rpl13* reverse: GGTACTTCCACCCGACCTC

*MT-ATP6* forward: ACACCCCTTATCCCCATACTAG

*MT-ATP6* reverse: ATGGTTGATATTGCTAGGGTGG

*RPL13* forward: CAAACTCATCCTCTTCCCCAG

*RPL13* reverse: CTCCTTCTTATAGACGTTCCGG

*ACTB* forward: CACCATTGGCAATGAGCGGTTC

*ACTB* reverse: AGGTCTTTGCGGATGTCCACGT

### Immunoprecipitation assay

Immunoprecipitation assays were performed according to a previous study. CCC-HEH-2 cells were collected and suspended in lysis buffer (pH 7.5): 50 mM Tris-HCL, 150 mM NaCl, 1 mM EDTA, 1% NP-40; protease inhibitors (Cell Signaling Technology, 5871) were added before employed. The lysate was immunoprecipitated with 20 μL protein A/G Plus-agarose (Santa Cruz Biotechnology, sc-2003) and primary antibodies at 4°C overnight. Finally, the precipitated proteins together with initial whole-cell lysates were subjected to western blots, as described above.

### Flow cytometry analysis

For the PI and ANXA5 staining assay, cells were harvested after drug treatment or transfection as indicated and washed with PBS. Then cells were placed in tubes and stained with FITC Annexin V Apoptosis Detection Kit I (BD Pharmingen, 556547) according to the manufacturer´s protocol.

For the mitochondrial membrane potential assay, a JC-1 probe (5 μM; Sigma-Aldrich, T4069) was used to measure mitochondrial depolarization in CCC-HEH-2 cells. After treatment, cells were digested with trypsin and incubated with an equal volume of JC-1 solution (5 μg/mL) at 37°C for 20 min in the dark. After washing, resuspend cells with 1× buffer.

A FACSCalibur cytometer (BD Biosciences, USA) was employed for analysis.

### Plasmid construction

Preparation of linearized vector: pLV242 was digested by restriction endonuclease KpnI and BamHI to obtain a single linearized fragment. The enzyme digestion system consisted of 2 μL 10× CutSmart buffer (New England BioLabs, B7204), 1 μg pLV242 vector, 1 μL KpnI (New England BioLabs, R3142) and BamHI (New England BioLabs, R0136) enzyme, then filled distilled water to 20 μL. Enzyme digest for 4 h at 37°C. The single linearized fragment was obtained with agarose gel electrophoresis. After the bands were separated to an appropriate interval, the target bands were cut with a tool knife for cleaning and recycling to obtain a single linearized carrier fragment.

Acquisition of insert fragments: Primers were designed according to the mutation sites, and high-fidelity DNA polymerase (New England BioLabs, M0492S) was used for PCR amplification. The reaction system was prepared according to the manufacturer’s protocol.

PCR reaction procedure: After denaturation at 95°C for 10 s, annealing was performed for 30 s, then the extension was performed at 68°C. The reaction was performed for 30 cycles. Then extend at 68°C for 10 min. Finally, the temperature drops to 4°C.

Transformation and sequencing: 10 μL recombinant product were added with Trans 5α (TransGen Biotech, CD201–01) to the 50 μL competent at 4°C for 30 min. Then immediately placed in a 42 thermostatic water bath for 45 s, and quickly removed and kept in ice for 1–2 m. Then added 1 mL of nonresistant medium. Shake at 37°C for 1 h. Coat the plate, and place it in an incubator for growth at 37°C overnight. The next day, single colonies were selected and added to the corresponding resistance medium to amplify the bacterial solution. Finally, the samples were sequenced and compared to confirm the single-point mutation. The primer sequences were as follows:

*H. sapiens* PRKAA^T172A^ Forward: ATTTTTAAGAGCCAGTTGTGGCTC
*H. sapiens* PRKAA^T172A^ Reverse: GCCACAACTGGCTCTTAAAAATTC
*H. sapiens* PRKAA^T172D^ Forward: TTAAGAGACAGTTGTGGCTCACC
*H. sapiens* PRKAA^T172D^ Reverse: AGCCACAACTGTCTCTTAAAAATTC
*H. sapiens* PRKAA^S485A^ Forward: GAGATCGGGAGCCGTTAGCAAC
*H. sapiens* PRKAA^S485A^ Reverse: GTTGCTAACGGCTCCCGATCTC
*H. sapiens* PRKAA^S485D^ Forward: GAGATCGGGAGACGTTAGCAAC
*H. sapiens* PRKAA^S485D^ Reverse: GTTGCTAACGTCTCCCGATCTC
*H. sapiens* pLV242-PRKAA Forward:

AAGGAATTCGGTACCATGCGCAGACTCAGTTCCTGG

*H. sapiens* pLV242-PRKAA Reverse:

TTTGTAGTCGGATCCTTGTGCAAGAATTTTAATTAGATTTG

### Statistical analysis

Results were expressed as the mean value ± standard deviation (SD). *P* value was calculated with one-way ANOVA or Student’s ttest (unpaired, two-tailed). The methods for multiple comparisons test were specified in figure legends. *P* < 0.05 was considered statistically significant. GraphPad Prism9 software was used for statistical analysis.

## Supplementary Material

Supplemental Material

Supplemental Material

## Data Availability

All data associated with this study are present in the paper or the Supplementary Materials. The data, methods for data analysis and related materials are available from the corresponding author on reasonable request. The RNA-Sequencing data was available in NCBI’s Gene Expression Omnibus database (GSE200325).
