# Supplementary material for: Inhibition of PRKAA/AMPK (Ser485/491) phosphorylation by crizotinib induces cardiotoxicity via perturbing autophagosome-lysosome fusion
Source: Autophagy. 2023 Sep 21;20(2):416–36. doi: 10.1080/15548627.2023.2259216 (PMC10813574; doi:10.1080/15548627.2023.2259216)
Supplement: Supplemental Material [file KAUP_A_2259216_SM9179.docx]

**Supplementary Material**

**Inhibition of PRKAA/AMPK (Ser485/491) Phosphorylation by Crizotinib Induces Cardiotoxicity via Perturbing Autophagosome-Lysosome Fusion**

Zhifei Xu^1^, Zezheng Pan^1^, Ying Jin^1^, Zizheng Gao^1^, Feng Jiang^1^, Huangxi Fu^1^, Xueqin Chen^2,3^, Xiaochen Zhang^4^, Hao Yan^1^, Xiaochun Yang^1^, Bo Yang^5^, Qiaojun He^1,6,7^ and Peihua Luo^1,6,8,*^

^1^Center for Drug Safety Evaluation and Research of Zhejiang University, College of Pharmaceutical Sciences, Zhejiang University, Hangzhou 310058, Zhejiang, P.R.China.

^2^Department of Oncology, Affiliated Hangzhou Cancer Hospital, Zhejiang University School of Medicine, Key Laboratory of Clinical Cancer Pharmacology and Toxicology Research of Zhejiang Province, Hangzhou 310002, Zhejiang, P.R.China.

^3^Cancer Center, Zhejiang University, Hangzhou 310058, Zhejiang, P.R.China.

^4^Department of Medical Oncology, The First Affiliated Hospital, Zhejiang University School of Medicine, Hangzhou 310003, Zhejiang, P.R.China.

^5^Institute of Pharmacology & Toxicology, College of Pharmaceutical Sciences, Zhejiang University, Hangzhou 310058, Zhejiang, P.R.China.

^6^Department of Cardiology, Second Affiliated Hospital, School of Medicine, Zhejiang University, Hangzhou 310009, Zhejiang, P.R.China.

^7^Innovation Institute for Artificial Intelligence in Medicine of Zhejiang University, Hangzhou 310018, Zhejiang, P.R.China.

^8^Department of Pharmacology and Toxicology, Hangzhou Institute of Innovative Medicine, College of Pharmaceutical Sciences, Zhejiang University, Hangzhou, 310018, P.R.China.

**Contact** Peihua Luo

peihualuo@zju.edu.cn

Center for Drug Safety Evaluation and Research of Zhejiang University, College of Pharmaceutical Sciences, Zhejiang University

866 Yuhangtang Road, Zhejiang University, Hangzhou, Zhejiang, P.R.China

Supplementary Figures 1-9 and Figure Legends


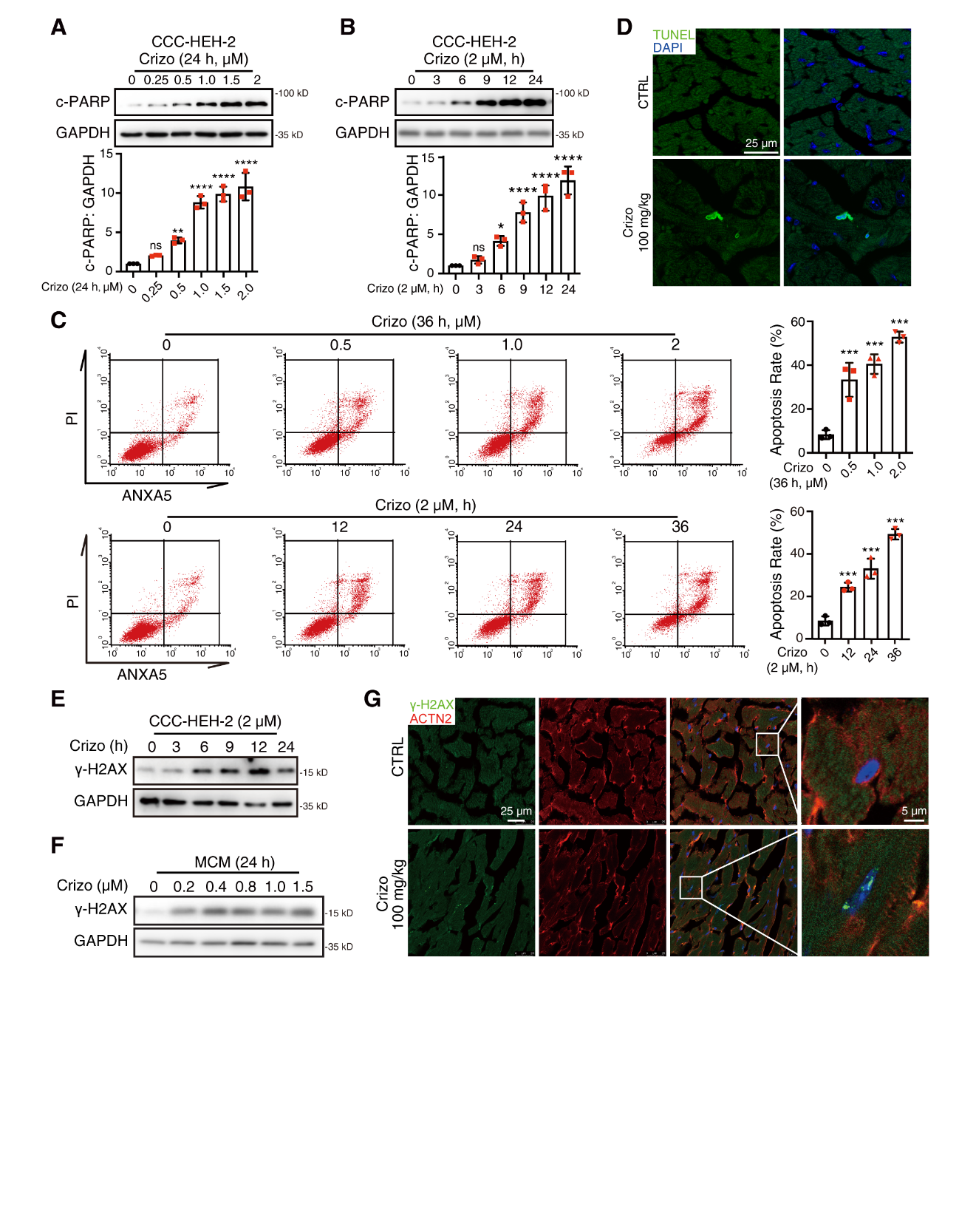


Figure S1. Crizotinib causes cardiomyocyte apoptosis and DNA damage. (A, B) CCC-HEH-2 cells were treated with crizotinib. GAPDH was used as a loading control. Representative images (upper) and relative quantification (lower) were shown. *n* = 3. (C) CCC-HEH-2 cells were treated with crizotinib. Then, cells were harvested and stained with PI and ANXA5/annexin V. The apoptosis rates were detected by flow cytometry. Representative images were shown on the left and a statistical histogram was presented on the right. *n* = 3. (D) Heart tissues from indicated group of mice were collected and the TUNEL signal was detected by immunofluorescence assay. Cardiac sections were stained by TUNEL (green) and DAPI (blue). Scale bar: 25 μm. (E, F) CCC-HEH-2 and MCMs were treated with crizotinib as indicated. Representative images of γ-H2AX immunoblots were shown. (G) Heart tissues from indicated group of mice were collected and representative images of cardiac sections stained by γ-H2AX (green) were shown. Nucleus was stained by DAPI and cardiomyocytes were stained with ACTN2/α-actinin. Scale bar: 25 μm. Enlarged views were shown on the right. Scale bar: 5 μm. Data were presented as mean ± SD. The *P* value was calculated by one-way ANOVA with Dunnett’s multiple comparisons tests (A-C). ***, *P* < 0.001; **, *P* < 0.01; *, *P* < 0.05; ns, no significance. γ-H2AX: phosphorylation of the serine residue of H2A.X variant histone; CTRL: control; Crizo: crizotinib.


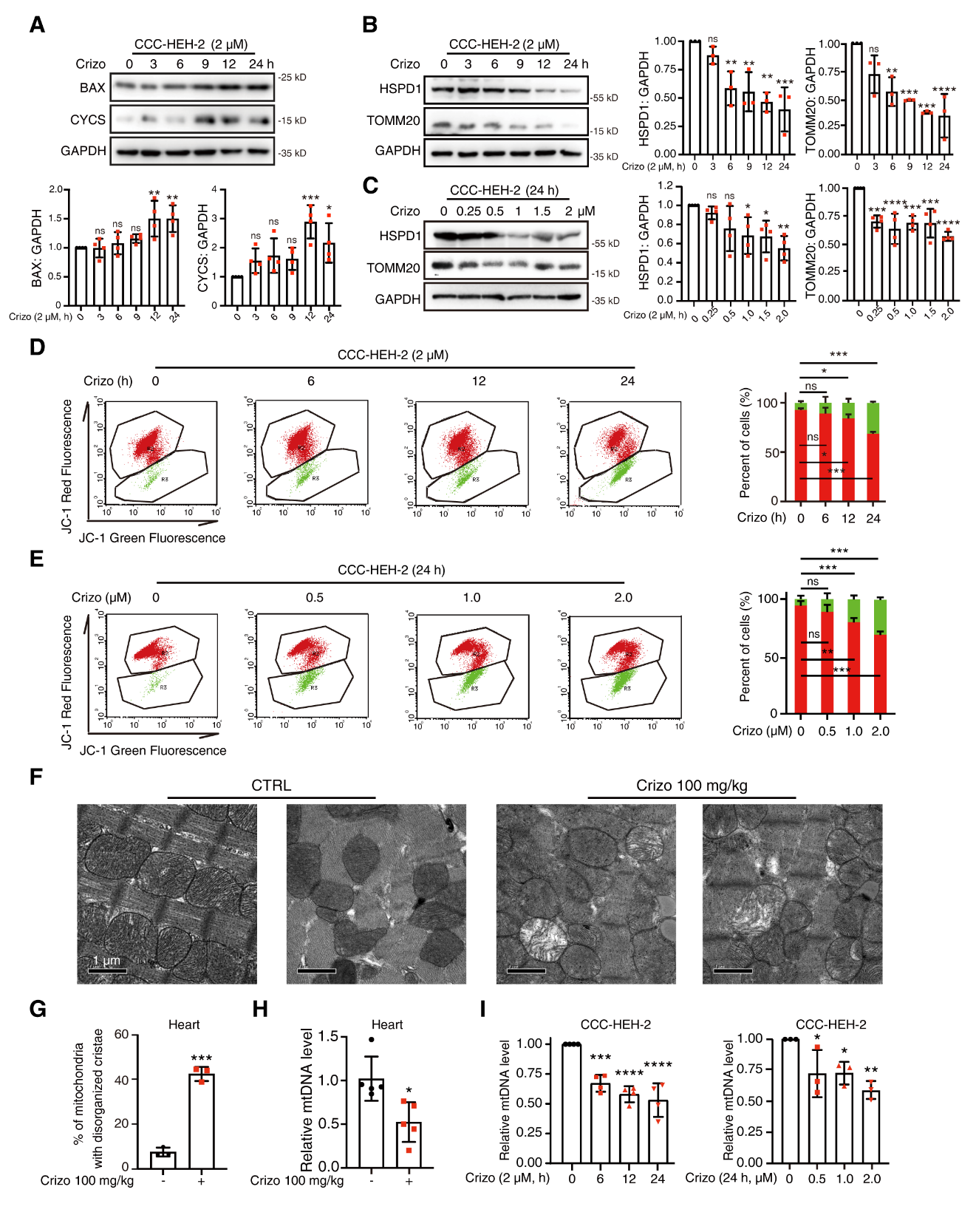


**Figure S2.** Crizotinib induces mitochondrial injury in cardiomyocytes. (**A**-**C**) Representative immunoblots (upper) and relative quantification (lower) of mitochondrial-related apoptosis and membrane protein levels. CCC-HEH-2 cells were treated with crizotinib as indicated. GAPDH was used as a loading control. *n* ≥ 3. (**D**, **E**) CCC-HEH-2 cells were treated with crizotinib. Then, cells were harvested and stained with JC-1, the red and green relative mean fluorescence values were detected by flow cytometry. *n* = 4. Representative images were shown on the left and statistical histograms were presented on the right. (**F**) Transmission electron microscopy observation of the left ventricle from mice from indicated groups and representative images were shown. Scale bar: 2 μm. (**G**) The percentage of mitochondria with disorganized cristae was analyzed with Image J software and a statistical histogram was shown. *n* = 3 hearts. (**H**, **I**) Total RNA was extracted from crizotinib-treated mice hearts and CCC-HEH-2 cells, and mRNA level was measured by qPCR. The *MT-ATP6* (mitochondria-encoded DNA): *RPL13* (nucleus-encoded DNA) ratio was indicated as relative mtDNA level. *n* = 5, 4, 3. Data were presented as mean ± SD. The *P* value was calculated by Student’s t test (**G** and **H**) and one-way ANOVA with Dunnett’s multiple comparisons test (**A**-**E** and **I**). ****, *P* < 0.0001; ***, *P* < 0.001; **, *P* < 0.01; *, *P* < 0.05; ns, no significance. CTRL: control; Crizo: crizotinib; mtDNA: mitochondrial DNA.


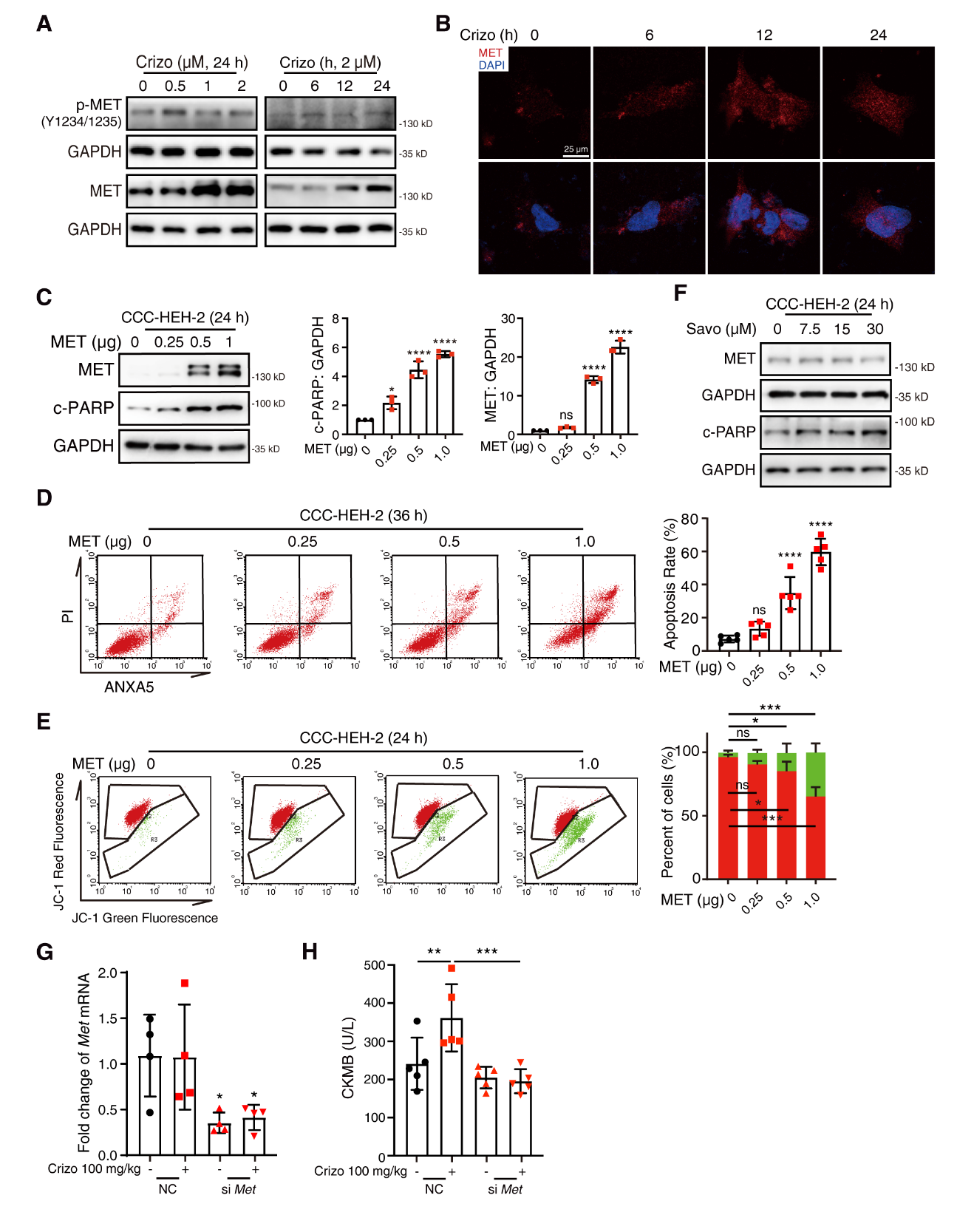


**Figure S3.** Accumulated MET contributes to cardiomyocyte death and mitochondrial injury. (**A**) Representative immunoblots of p-MET (Y1234/1235). CCC-HEH-2 cells were treated with crizotinib as indicated. GAPDH was used as a loading control. (**B**) Representative immunofluorescence images of MET staining in CCC-HEH-2 cells treated with crizotinib. Scale bar: 25 μm. (**C**) CCC-HEH-2 cells were transfected with 0, 0.25, 0.5 or 1 μg pCMV3-MET plasmid. 24 h after transfection, total cell lysates were used to detect the expression levels of MET and c-PARP. GAPDH was used as a loading control. The quantification results were shown on the right. *n* = 3. (**D**, **E**) CCC-HEH-2 cells were transfected with 0, 0.25, 0.5 or 1 μg pCMV3-MET plasmid. 24 h after transfection, cells were harvested and stained with PI and ANXA5 or JC-1. The apoptosis rates (*n* = 5) or relative mean fluorescence values of red and green were detected by flow cytometry. Representative images were shown on the left and a statistical histogram was presented on the right. (**F**) CCC-HEH-2 cells were treated with savolitinib for 24 h. GAPDH was used as a loading control. (**G**, **H**) AAV9-*TNNT2*-si *Met* or AAV9-*TNNT2*-NC virus was injected into C57BL/6J mice through the tail vein. Three weeks after injection, mice were then intragastrically administrated with vehicle or 100 mg/kg crizotinib for another 6 weeks. (**G**) Total RNA was extracted from mice hearts (*n* = 4) and *Met* mRNA level relative to *Actb* was measured by qPCR. (**H**) Serum from indicated mice analyzed for CKMB level. *n* = 5. Data were presented as mean ± SD. The *P* value was calculated by one-way ANOVA with Dunnett’s (**C**-**E**), Fisher’s LSD (**G**) or Sidak’s test (**H**) multiple comparisons tests. ****, *P* < 0.0001; ***, *P* < 0.001; **, *P* < 0.01; *, *P* < 0.05; ns, no significance. Savo: savolitinib.


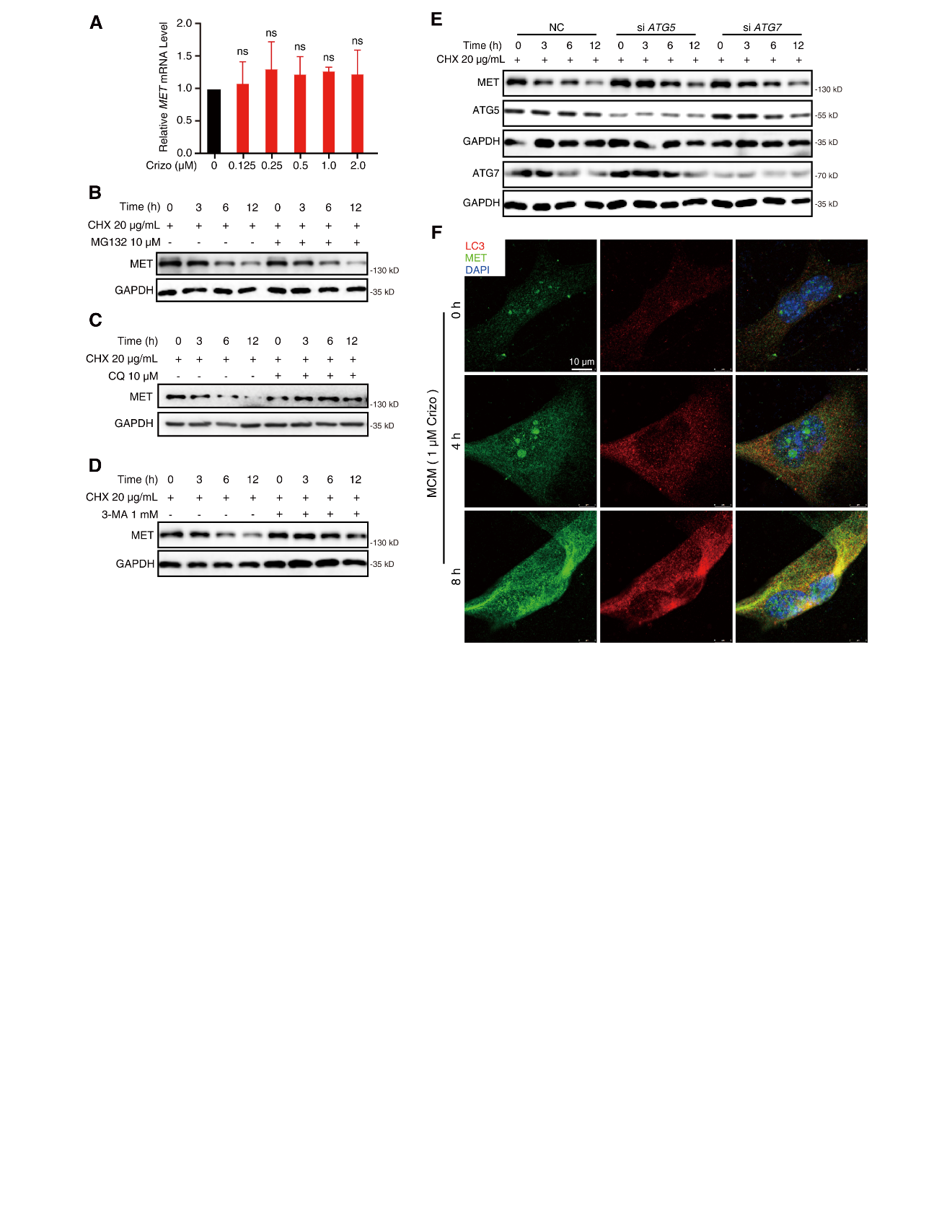


**Figure S4.** Crizotinib prevents the autophagic degradation of MET. (**A**) CCC-HEH-2 cells were treated with crizotinib (0, 0.25, 0.5, 1, 1.5 or 2 μM) for 12 h. Then cells were harvested for total RNA extraction and the relative *MET* mRNA level to *ACTB* was measured by qPCR. *n* = 3. (**B**-**E**) Representative immunoblots of MET. GAPDH was used as a loading control. (**B**) CCC-HEH-2 cells were treated with CHX (20 μg/mL) with or without MG132 (10 μM) for 0, 3, 6 or 12 h. (**C**) CCC-HEH-2 cells were treated with CHX (20 μg/mL) with or without CQ (10 μM) for 0, 3, 6 or 12 h. (**D**) CCC-HEH-2 cells were treated with CHX (20 μg/mL) with or without 3-MA (1 mM) for 0, 3, 6 or 12 h. (**E**) CCC-HEH-2 cells were transfected with siRNA targeting *ATG5* and *ATG7*, and then treated with CHX (20 μg/mL) for 0, 3, 6 or 12 h. (**F**) The co-localization of LC3 with MET in untreated or crizotinib-treated (1 μM) MCMs. The cells were stained for LC3 (red), MET (green) and DAPI (blue). Scale bar: 20 μm. Data were presented as mean ± SD. The *P* value was calculated by one-way ANOVA with Dunnett’s multiple comparisons test. ns, no significance. Crizo: crizotinib.


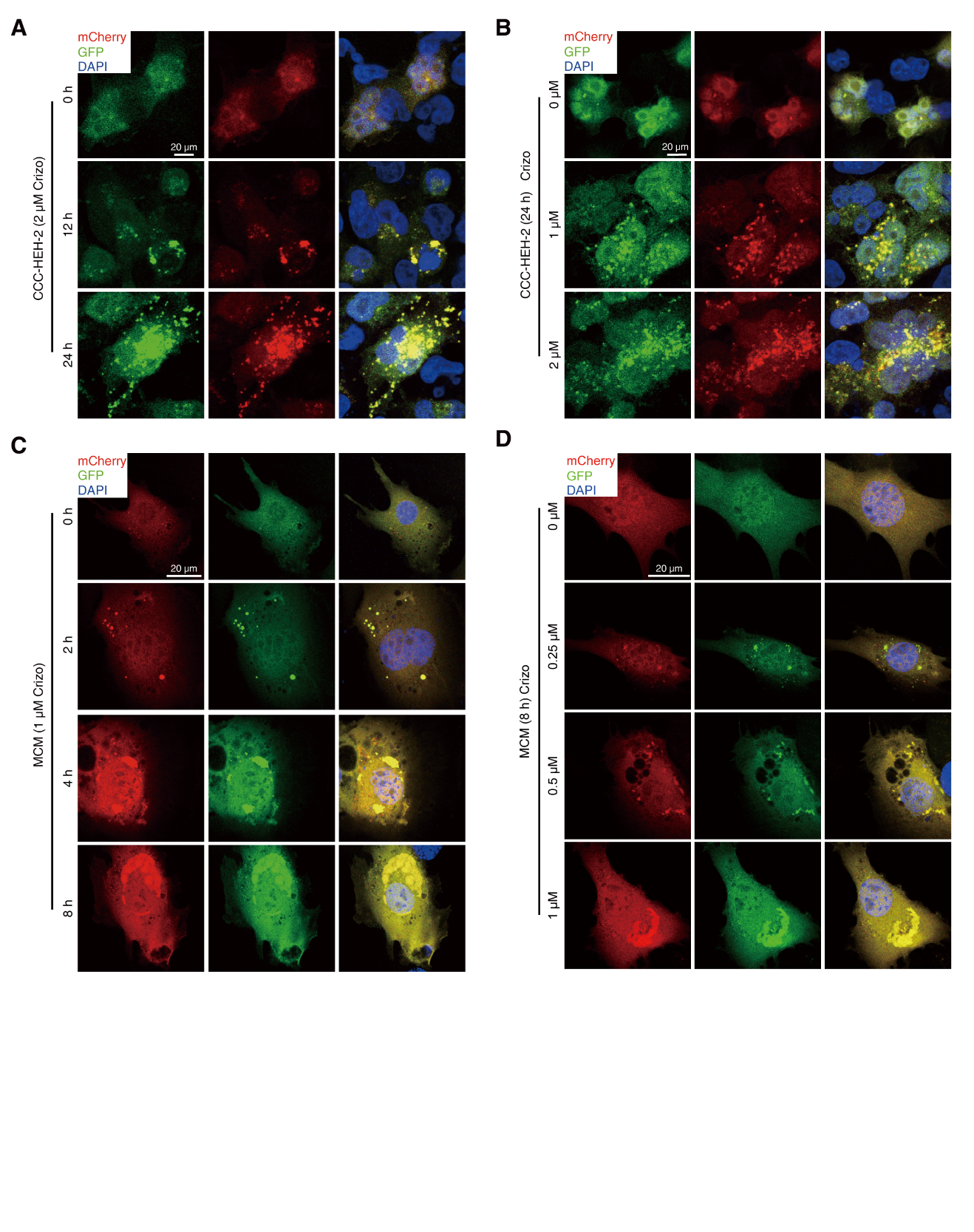


**Figure S5.** Crizotinib inhibits autophagosome-lysosome fusion in cardiomyocytes. Autophagic flux assays were performed by confocal microscopy. (**A**, **B**) CCC-HEH-2 cells were infected with mCherry-GFP-LC3 virus for 12 h and treated with crizotinib (2 μM) for 0, 12, 24 h or 0, 1, 2 μM for 24 h. Scale bar: 20 μm. (**C**, **D**) MCMs were infected with mCherry-GFP-LC3 virus for 12 h and treated with crizotinib (1 μM) for 0, 2, 4, 8 h or 0, 0.25, 0.5, 1 μM for 8 h. Scale bar: 20 μm. Crizo: crizotinib.


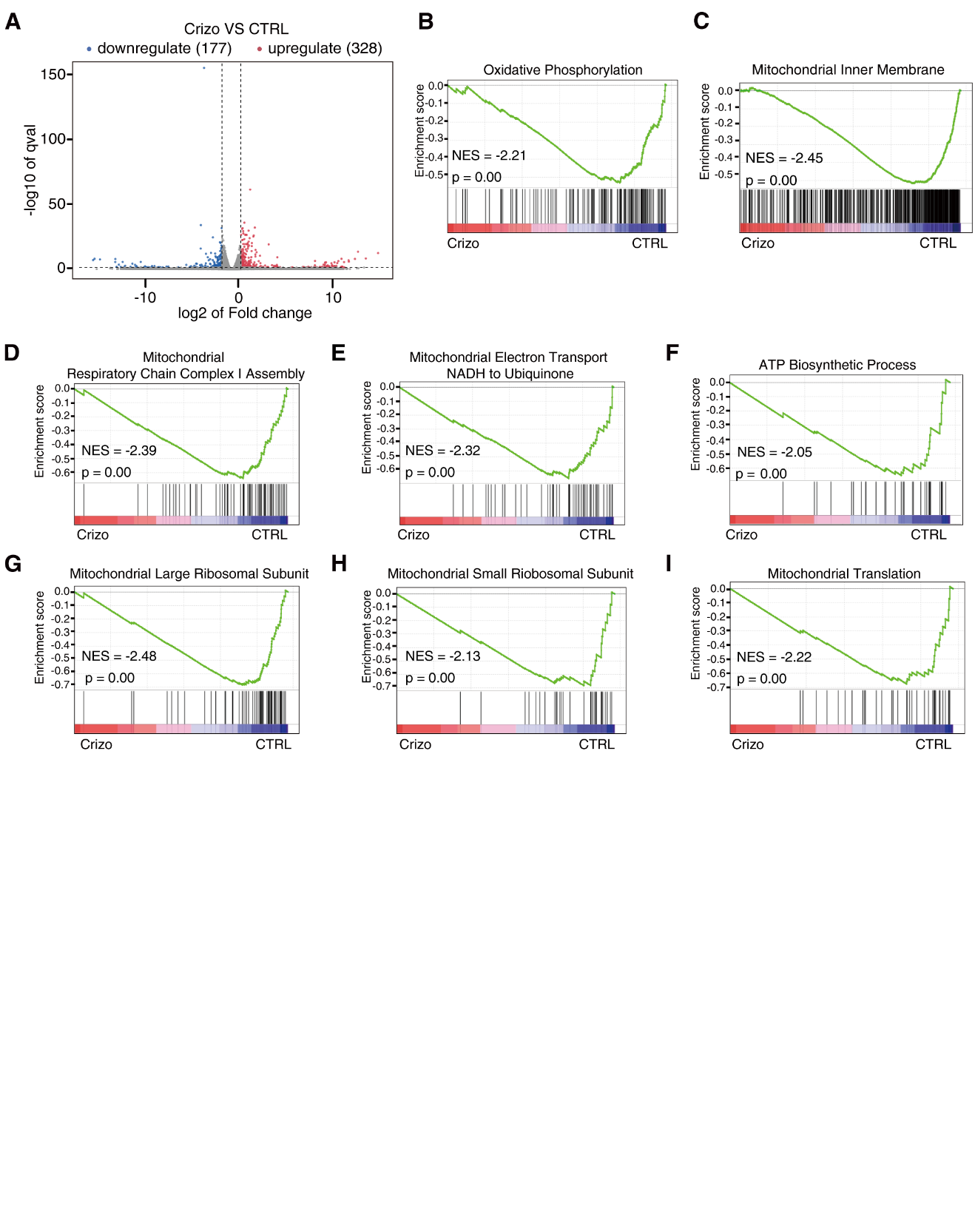


**Figure S6.** Crizotinib regulates the expression of genes related to autophagy and mitochondrial homeostasis. CCC-HEH-2 cells were treated with or without 2 μM crizotinib for 12 h and subjected to RNA-Sequencing analysis. (**A**) Volcano plot of differentially expressed genes between the control and crizotinib-treated CCC-HEH-2 cells. (**B**-**I**) Gene Set Enrichment Analysis (GSEA) on specific pathways as indicated. CTRL: control; Crizo: crizotinib; NES: Normalized enrichment score.


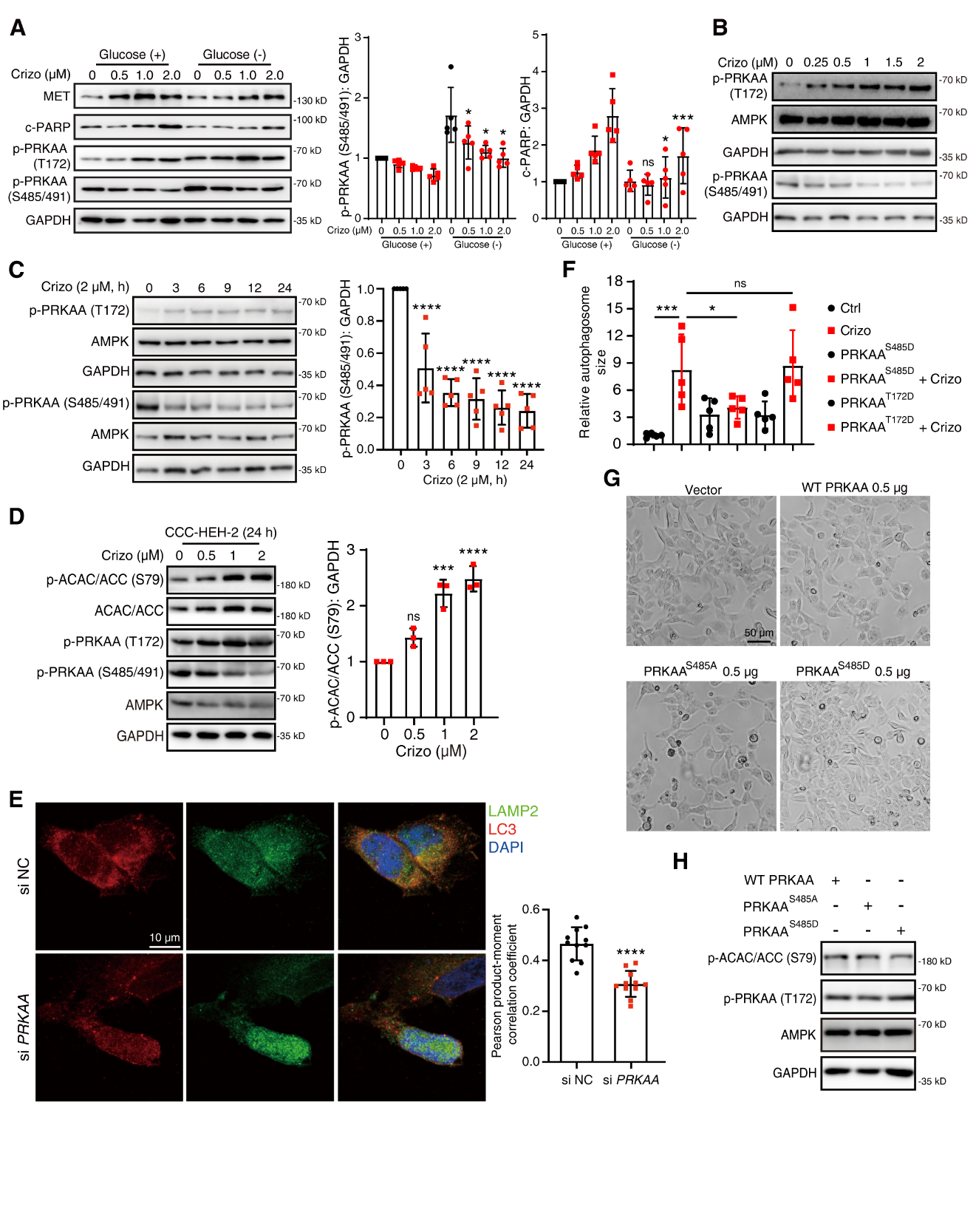


**Figure S7.** Crizotinib inhibits PRKAA (Ser485/491) phosphorylation. (**A**) CCC-HEH-2 cells were treated with crizotinib for 24 h and with or without 4 h glucose-starvation administration, respectively. Representative immunoblots were shown. GAPDH was used as a loading control. *n* = 5. (**B**-**D**) CCC-HEH-2 cells were treated with crizotinib. Representative immunoblots were shown. GAPDH was used as a loading control. (**E**) CCC-HEH-2 cells were transfected with siRNA targeting *PRKAA* or negative control, and then treated with serum starvation stress for 2 h. The cells were stained for LC3 (red), LAMP2 (green) and DAPI (blue). Scale bar: 10 μm. Pearson correlation coefficients were analyzed as LAMP2 and LC3 co-localization rates. *n* = 11 fields from more than three independent experiments per group. (**F**) CCC-HEH-2 cells were infected with the mCherry-GFP-LC3 virus. 12 h after infection, cells were transfected with PRKAA^S485D^ and PRKAA^T172D^ plasmid, respectively. Then 6 h after transfection, cells were treated with crizotinib (2 μM, 24 h). Autophagic flux assays were performed with the confocal microscope. Quantification of the size of autophagosome puncta was shown. *n* = 5 per group. (**G**) CCC-HEH-2 cells were transfected with 0.5 μg of Vector, WT PRKAA, PRKAA^S485A^ and PRKAA^S485D^ plasmid respectively. Representative images of cell morphology were obtained by microscope. Scale bar: 50 μm. (**H**) CCC-HEH-2 cells were transfected with 0.5 μg of WT PRKAA, PRKAA^S485A^ and PRKAA^S485D^ plasmid respectively. Representative immunoblots were shown. GAPDH was used as a loading control. The *P* value was calculated by one-way ANOVA with Dunnett’s (**C, D**), Sidak`s multiple comparisons tests (**F**) or Student’s t test (**E**). ****, *P* < 0.0001; ***, *P* < 0.001; *, *P* < 0.05; ns, no significance. CTRL: control; Crizo: crizotinib.


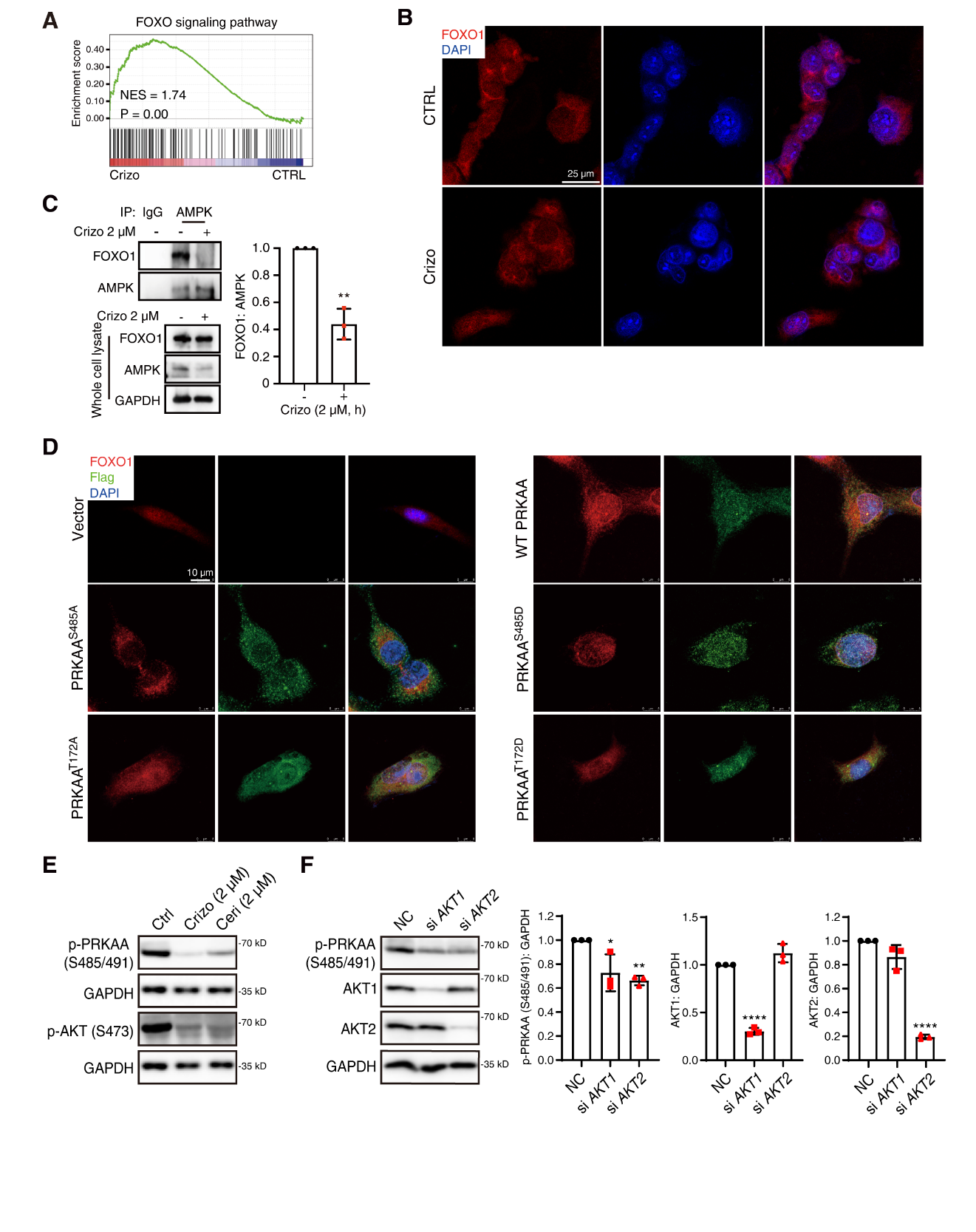


**Figure S8.** Crizotinib inhibits AKT-AMPK-FOXO1 signaling pathways. (**A**) CCC-HEH-2 cells were treated with or without 2 μM crizotinib for 12 h and 3 independent repeated samples were subjected to RNA-Seq analysis. Gene set enrichment analysis (GSEA) of the FOXO1 signaling pathway was shown. (**B**) CCC-HEH-2 cells were treated with 2 μM crizotinib for 24 h. Immunofluorescence assay for FOXO1 and nucleus (DAPI). Representative images of FOXO1 subcellular localization. Scale bar: 25 μm. (**C**) CCC-HEH-2 cells were treated with crizotinib (2 μM) and co-immunoprecipitation was performed with an anti-AMPK antibody. Both cell lysates and immunoprecipitates were subjected to western blot. Representative images (left) and relative quantification (right) were shown. The experiments were performed three times independently. (**D**) CCC-HEH-2 cells were transfected with WT PRKAA, PRKAA^S485A^, PRKAA^S485D^, PRKAA^T172A^ and PRKAA^T172D^ plasmids, respectively. Representative images of cells stained by FOXO1 and Flag (PRKAA) were captured with a confocal microscope. Scale bar: 10 μm. (**E**) CCC-HEH-2 cells were treated with 2 μM crizotinib or ceritinib for 24 h. Representative immunoblots were shown. GAPDH was used as a loading control. *n* = 3. (**F**) CCC-HEH-2 cells were transfected with siRNA targeting *AKT1* or *AKT2*. Representative immunoblots (left) and relative quantification (right) were shown. GAPDH was used as a loading control. *n* = 3. Data were presented as mean ± SD. The *P* value was calculated by Student’s t test (**C**) or one-way ANOVA with Dunnett’s multiple comparisons tests (**F**). ****, *P* < 0.0001; **, *P* < 0.01; *, *P* < 0.05. CTRL: control; Crizo: crizotinib; Ceri: ceritinib.


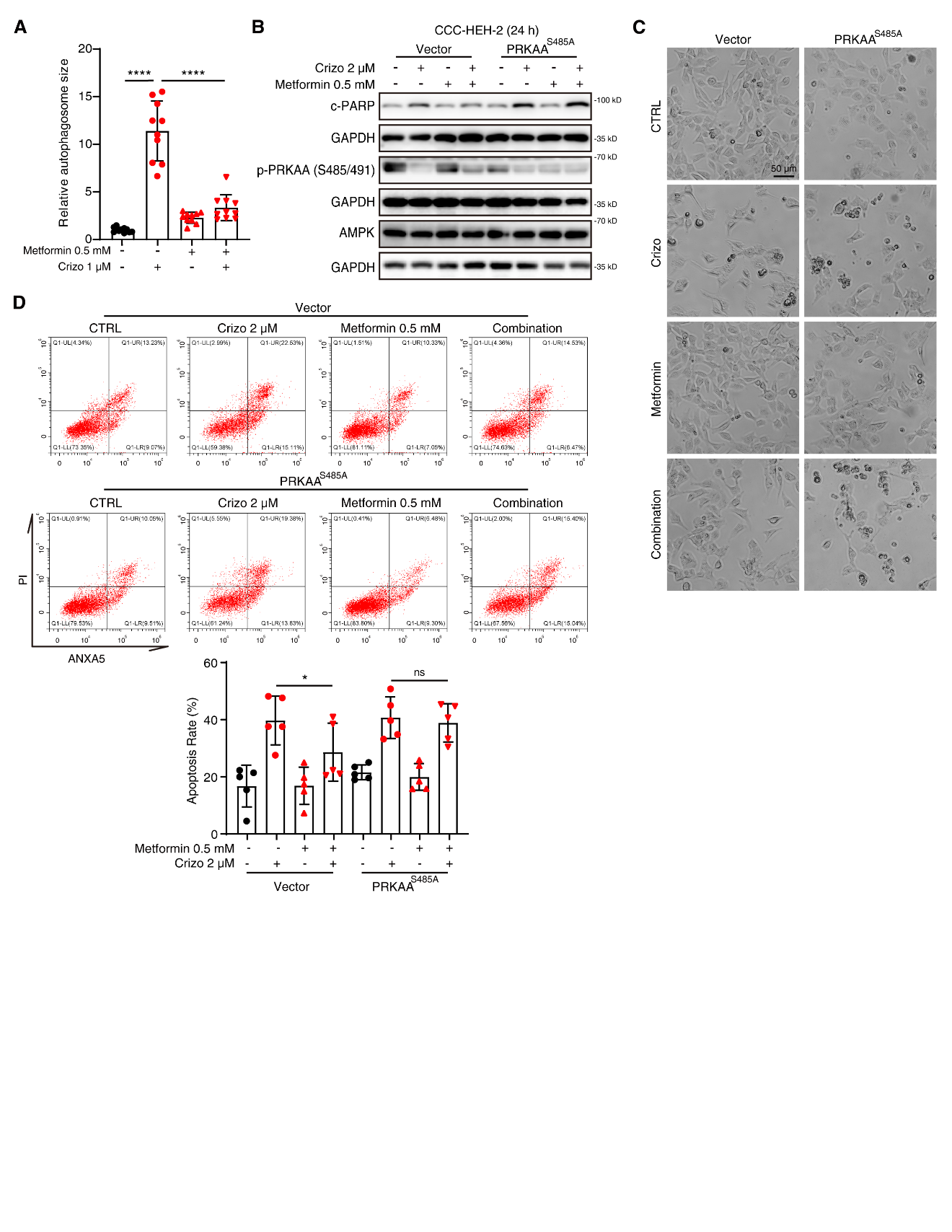


**Figure S9.** Metformin protects cardiomyocytes from crizotinib-induced apoptosis by recovering PRKAA (Ser485/491) phosphorylation. (**A**) MCMs were infected with the mCherry-GFP-LC3 virus. 12 h after infection, cells were treated with crizotinib with or without metformin. Autophagic flux assays were performed with the confocal microscope. Quantification of the size of mCherry and GFP fluorescent puncta was shown. *n* = 10 fields per group. (**B-D**) CCC-HEH-2 cells were transfected with Vector or PRKAA^S485A^ plasmids and then treated with crizotinib (2 μM) with or without metformin (0.5 mM). (**B**) Representative immunoblots of c-PARP, p-PRKAA (S485/491) and PRKAA were shown. GAPDH was used as a loading control. (**C**) Representative images of cell morphology were obtained by microscope. Scale bar: 50 μm. (**D**) Cells were harvested and stained with PI and ANXA5, and apoptosis rates were detected by flow cytometry. *n* = 5. Representative images were shown on the left and a statistical histogram was presented beneath. Data were presented as mean ± SD. The *P* value was calculated by one-way ANOVA with Sidak’s test. ****, *P* < 0.0001; *, *P* < 0.05; ns, no significance. CTRL: control; Crizo: crizotinib.
